# Antibiotic resistance, plasmids, and virulence-associated markers in human strains of *Campylobacter jejuni* and *Campylobacter coli* isolated in Italy

**DOI:** 10.3389/fmicb.2023.1293666

**Published:** 2024-01-08

**Authors:** Aurora Garcia-Fernandez, Anna Janowicz, Francesca Marotta, Maira Napoleoni, Sergio Arena, Sara Primavilla, Monica Pitti, Romina Romantini, Fiorella Tomei, Giuliano Garofolo, Laura Villa

**Affiliations:** ^1^Department of Infectious Diseases, Istituto Superiore di Sanità, Rome, Italy; ^2^National Reference Laboratory for Campylobacter, Istituto Zooprofilattico Sperimentale dell'Abruzzo e del Molise "G. Caporale", Teramo, Italy; ^3^Centro di Riferimento Regionale Patogeni Enterici, CRRPE, Istituto Zooprofilattico Sperimentale dell’Umbria e delle Marche “T. Rosati”, Perugia, Italy; ^4^Centro di Riferimento per la Tipizzazione delle Salmonelle, CeRTiS, Istituto Zooprofilattico Sperimentale del Piemonte Liguria e Valle d'Aosta, Turin, Italy; ^5^BIOS Laboratory, Rome, Italy

**Keywords:** *Campylobacter*, cgMLST, virulence, pTet, pVir, tet(O), antibiotic resistance, GyrA

## Abstract

Campylobacteriosis, a prevalent foodborne gastrointestinal infection in Europe, is primarily caused by *Campylobacter jejuni* and *Campylobacter coli*, with rising global concerns over antimicrobial resistance in these species. This study comprehensively investigates 133 human-origin *Campylobacter* spp. strains (102 *C. jejuni* and 31 *C. coli*) collected in Italy from 2013 to 2021. The predominant Multilocus Sequence Typing Clonal complexes (CCs) were ST-21 CC and ST-206 CC in *C. jejuni* and ST-828 CC in *C. coli*. Ciprofloxacin and tetracycline resistance, mainly attributed to GyrA (T86I) mutation and *tet*(O) presence, were prevalent, while erythromycin resistance was associated with 23S rRNA gene mutation (A2075G), particularly in *C. coli* exhibiting multidrug-resistant pattern CipTE. Notable disparities in virulence factors among strains were observed, with *C. jejuni* exhibiting a higher abundance compared to *C. coli*. Notably, specific *C. jejuni* sequence types, including ST-21, ST-5018, and ST-1263, demonstrated significantly elevated counts of virulence genes. This finding underscores the significance of considering both the species and strain-level variations in virulence factor profiles, shedding light on potential differences in the pathogenicity and clinical outcomes associated with distinct *C. jejuni* lineages. *Campylobacter* spp. plasmids were classified into three groups comprising pVir-like and pTet-like plasmids families, exhibiting diversity among *Campylobacter* spp. The study underscores the importance of early detection through Whole Genome Sequencing to identify potential emergent virulence, resistance/virulence plasmids, and new antimicrobial resistance markers. This approach provides actionable public health data, supporting the development of robust surveillance programs in Italy.

## 1 Introduction

Campylobacteriosis has been the most frequently reported foodborne gastrointestinal infection in humans in the European Union (EU) since 2007. In 2021, the total number of confirmed human cases in the EU was 127,840, and *Campylobacter* spp. was the fourth most common cause of foodborne outbreaks reported by 17 Member States (MSs) and three non-MSs at EU level (EFSA and ECDC, 2022). The main *Campylobacter* species reported in EU was *Campylobacter jejuni* (88.4%), followed by *Campylobacter coli* (10.1%) (EFSA and ECDC, 2021). The incidence of campylobacteriosis varies globally between countries, and its true incidence remains uncertain due to underreporting of *Campylobacter* spp. infection cases, disparities in reporting systems, diagnosis challenges, and differences in outbreak surveillance (Hansson et al., 2018). Notifications of campylobacteriosis cases in Italy are gathered by the Enter-Net Italia surveillance and reported annually to the European Center for Disease Prevention and Control (ECDC). This surveillance was non-mandatory until 2022, leading to underestimating the annual number of reported cases. The iceberg effect is well known, and a population-based serological study indicated Italy as a country with a low reporting rate in 2009–2013 (Cassini et al., 2018). Despite the parsimonious collection of strains in Italy, it remains crucial to determine the main characteristics of the circulating strains through their complete characterization. *Campylobacter* spp. is primarily found in the digestive tract of poultry and poultry meat, as well as other animals and food matrices thereof such as cattle and swine (Wysok et al., 2015). Contact with dogs and cats can also be a risk factor for human campylobacteriosis (Andrzejewska et al., 2013).

Campylobacteriosis is typically self-limiting and usually resolves within a week from the onset of symptoms (Blaser and Engberg, 2008). Antimicrobial treatment should be reserved for cases of severe gastroenteritis, extraintestinal infections, or immunocompromised patients. Nonetheless, the rising antibiotic resistance (AMR) of *Campylobacter* spp. has emerged as a global issue (Skirrow, 1994; Dai et al., 2020). According to the European data, very high to extremely high levels of resistance to ciprofloxacin in *C. jejuni* and *C. coli* and high and very high levels of tetracycline resistance, respectively, in *C. jejuni* and *C. coli* were reported in humans (EFSA and ECDC, 2023). While, erythromycin resistance for *C. jejuni* was either absent or detected at very low levels, *C. coli* exhibited higher resistance levels. Low levels of gentamicin resistance were observed in *C. coli.* Combined resistance to ciprofloxacin and erythromycin, critical antimicrobials for the campylobacteriosis treatment, was generally uncommon in *C. jejuni* and moderately common in *C. coli* (EFSA and ECDC, 2023).

The main mechanism causing quinolone and fluoroquinolone resistance in *Campylobacter* spp. is the C257T point mutation in *gyrA* that yields a Thr-86-Ile amino acid change. Mutations in the 23S rRNA genes, amino acid changes in L4/L22 ribosomal proteins, and the presence of the ribosomal methylase encoded by *ermB* gene confer resistance to macrolides. The *tet*(O) gene encoding a ribosomal protection protein is mainly responsible for tetracycline resistance (Connell et al., 2003; Vacher et al., 2005; Iovine, 2013; Panzenhagen et al., 2021; Bunduruș et al., 2023).

Multilocus sequence typing (MLST) is a gold standard informative tool to analyze the molecular epidemiology of *Campylobacter* spp. allowing the characterization of the population structure of *Campylobacter* spp. and the identification of lineages such as sequence types (STs) and clonal complexes (CCs) (Dingle et al., 2001). Previous MLST studies on *C. jejuni* demonstrated that its population is genetically diverse and weakly clonal, consisting of large CCs representing epidemiologically relevant units for investigating *C. jejuni* epidemiology (Manning et al., 2003; Mouftah et al., 2021). Additionally, a higher level of discrimination is given by comparing the genomic sequences through the core genome multilocus sequence typing (cgMLST), which provides high-resolution data across related but not identical strains (Maiden et al., 2013; Cody et al., 2017; Hsu et al., 2020).

Specific *Campylobacter* spp. virulence genes encompassing its virulome represent relevant public health risks due to their capacity to strengthen the bacterium against the immunological response mounted by the host (Bunduruș et al., 2023). Most of the studies about the *Campylobacter* spp. virulome have been performed in *C. jejuni*, with fewer focused on *C. coli* (Fiedoruk et al., 2019; Bravo et al., 2021; Panzenhagen et al., 2021; Deblais et al., 2023; El-Adawy et al., 2023). Several *Campylobacter* spp. virulence factors have been linked to pathogenesis, severe illness and post-infection issues (Lapierre et al., 2016; Wieczorek et al., 2018). Utilizing flagella for motility, these bacteria navigate the mucus layer to reach and adhere to intestinal epithelial cells, a pivotal step in initiating infection. The ability to invade these cells, coupled with the production of toxins such as the cytolethal distending toxin (CDT), enhances the pathogenicity of the bacteria by inducing cell cycle arrest and apoptosis. Certain strains exhibit increased virulence through the presence of a protective polysaccharide capsule, while the microaerophilic nature of *Campylobacter* spp. enables them to thrive in the low-oxygen environment of the intestinal tract (Bunduruș et al., 2023; Kemper and Hensel, 2023). This intricate combination of virulence factors underscores the complexity of *Campylobacter* spp. pathogenesis, necessitating a comprehensive understanding of the development of effective preventive and therapeutic strategies.

Plasmids in *Campylobacter* spp., play a crucial role in shaping the pathogenicity and adaptability of these foodborne pathogens. Studies have identified various plasmid families, including pVir-like and pTet-like, each with particular replicon types or relaxases and potential implications for bacterial virulence and survival (van Vliet et al., 2021; Hull et al., 2023). Additionally, plasmids facilitate horizontal gene transfer, rapidly disseminating genetic material and traits, further enhancing the adaptability of *Campylobacter* spp. populations. The presence of plasmids carrying virulence factors underscores their significance in the pathogenic potential of these bacteria.

Understanding the mechanisms underlaying *Campylobacter* spp’s ability to acquire AMR, identifying virulence factors involved in the pathogenesis of campylobacteriosis, and studying plasmids carrying both genetic determinants are crucial in comprehending the infection mechanisms and the bacterium’s response to host immunity defense and antibiotic treatments.

This study employed whole genome sequencing (WGS) to comprehensively investigate the genetic diversity, antibiotic resistance, virulence content, and plasmid distribution in 133 *Campylobacter* spp. strains (102 *C. jejuni* and 31 *C. coli*) of human origin, collected in Italy from 2013 to 2021. This investigation integrated both resistome analysis and phenotypic approaches to assess antibiotic resistance, providing a comprehensive understanding of the genomic and phenotypic characteristics of these pathogens.

## 2 Materials and methods

### 2.1 Settings and bacterial strains

Surveillance for *Campylobacter* spp. in human strains in Italy is based on a systematic voluntary network (Enter-Net Italia) coordinated by the Infectious Disease Department of the *Istituto Superiore di Sanità* (ISS). The selection criteria in this study involved choosing epidemiologically unrelated viable strains from 2013 to 2021. Epidemiologically unrelated strains were defined as those isolated from patients without any known epidemiological link. This approach aimed to avoid possible submerged clonal events and assess the extent of clonal diversity during the chosen period in our country. One-hundred-thirty-three strains belonging to the two main notified species were selected: 102 *C. jejuni* and 31 *C. coli* (Supplementary Table S1), which were isolated from feces (128) and blood (5). These strains were received in swabs with already isolated and identified *C. jejuni* and *C. coli* strains. Subsequently, they were inoculated onto modified charcoal cefoperazone deoxycholate agar (mCCD Agar; Biolife Italiana srl, Milan, Italy) and Columbia Blood Agar Horse plates (Biolife Italiana srl, Milan, Italy). The plates were all incubated at 41.5°C ± 1°C for 48 ± 2 h in a microaerobic atmosphere and then screened for antibiotic susceptibility and DNA extraction.

### 2.2 Antibiotic susceptibility testing

Susceptibility was determined by the reference broth microdilution method and the Kirby-Bauer disk diffusion susceptibility test, following the international guideline recommendations of the European Committee on Antimicrobial Susceptibility Testing (EUCAST; www.eucast.org). Four antibiotics (Becton Dickinson, MD 21152–0999, United States) were tested by disk diffusion method, and the antibiotic concentrations were as follows: ciprofloxacin (Cip, 5 μg), tetracycline (T, 30 μg), erythromycin (E, 15 μg), and gentamicin (Gm, 10 μg). The control strain for antibiotics susceptibility testing was *C. jejuni* strain ATCC 33560. The susceptibility results were interpreted using the EUCAST guidelines.^1^ Broth microdilution method using Sensititre automated system (TREK Diagnostic Systems, Venice, Italy) was used to determine the antibiotic susceptibility of *Campylobacter* spp. strains. Cip, T, E, and Gm were tested according to previously reported methods (Marotta et al., 2020).

The strains were classified as resistant and susceptible according to minimum inhibitory concentration (MIC) breakpoints using Swin v3.3 Software (Thermo Fisher Scientific) following the epidemiological cutoff values (ECOFFs) as defined by EUCAST.^2^ MIC breakpoints of resistance applied were > 0.5 μg/mL for Cip (*C. jejuni* and *C. coli*), > 4 μg/mL for E (*C. jejuni*) and > 8 μg/mL (*C. coli*), > 2 μg/mL for Gm (*C. jejuni* and *C. coli*), and > 1 μg/mL for T (*C. jejuni*) and > 2 μg/mL (*C. coli*)*. Campylobacter jejuni* strain NCTC11351 was used as a control. Multidrug resistance (MDR) was defined as the resistance to three or more classes of antibiotics.

### 2.3 Whole genome sequencing

All the strains were sequenced by next-generation sequencing. Genomic DNAs were purified using the Macherey-Nagel NucleoSpin Tissue kit (Fisher Scientific Italia, Segrate, Italy). DNA libraries were created using Nextera XT Library Preparation Kit (Illumina, Inc., San Diego, CA, United States) and sequenced with Illumina NextSeq 500 sequencer, producing 150 bp paired-end reads.

Multiple online web-based bioinformatics tools (accessed September–December 2022) were used for pathogen characterization. Basic metrics and quality check of FASTQ-formatted sequencing reads were determined with FastQC Read Quality Reports v.0.72 (https://www.bioinformatics.babraham.ac.uk/projects/fastqc/; Galaxy-ARIES; Afgan et al., 2018; Knijn et al., 2020). The obtained raw reads were screened for contamination and reidentification of the species using Quality control species-identification pipelines KmerFinder v.3.2 for bacteria organisms (Center for Genomic Epidemiology, CGE; https://www.genomicepidemiology.org/; Hasman et al., 2014; Larsen et al., 2014; Clausen et al., 2018) and RefSeq Masher Matches v.0.1.2 (Galaxy-ARIES, https://w3.iss.it/site/aries/; Ondov et al., 2016; Knijn et al., 2020).

Quality trimming was performed by Trimmomatic v.0.38.1 to remove low-quality and adapter sequences for paired-end reads, filtering for a minimum read length of 50 and trimming low-quality 3′ ends of reads. Nucleotide positions in the reads with a quality score lower than Q20 were removed (Parameters: LEADING: 3; TRAILING: 3; SLIDINGWINDOW: 4:20; MINLEN:50) (Galaxy-ARIES; Bolger et al., 2014). *De novo* assembly of Illumina reads was performed using the SPAdes v.3.14.1 (Parameters: single-cell, with error correction and automatically k-mer values and coverage cuttoff) (Galaxy-ARIES; Bankevich et al., 2012; Afgan et al., 2018; Knijn et al., 2020). With the Staramr v.0.9.1 tool (Galaxy Europe, http://usegalaxy.eu/; Bharat et al., 2022), the genomes have been checked for genome sizes between the 1.6–1.9 Mbp range and the number of assembled contigs (Afgan et al., 2018; Bharat et al., 2022).

### 2.4 MLST and cgMLST analysis

Multilocus sequence typing scheme for *C. jejuni* and *C. coli* based on seven loci (a*spA, glnA, gltA, glyA, pgm, tkt, and uncA*) was performed by mlst 2.22.0 (Galaxy Europe; Bharat et al., 2022). Where STs were not defined, allele sequences were submitted to the *Campylobacter* spp. public database for molecular typing and microbial genome diversity.^3^ Based on their STs, strains were assigned to CCs using the *Campylobacter jejuni/coli* PubMLST.org database (Jolley et al., 2018).

Analysis by cgMLST assignment based on the 1,343 core loci using the *Campylobacter jejuni/coli* PubMLST.org database was conducted (Jolley et al., 2018). The cgMLST scheme used was described in Cody et al. (2017). The percentage of called targets ranged from 80.0 to 95.7% with an average of 92.5% of alleles called.

A dendrogram was generated based on cgMLST allelic differences, selecting the “Campylobacter (PubMLST) species” parameter. We used the Newick matrix created by the cgMLST Finder 1.2 v.1.0.1 method version 3.69 (CGE) to generate Neighbor-Joining tree (Clausen et al., 2018; Jolley et al., 2018). The tree was annotated using iTOL version 6 (Letunic and Bork, 2007). A minimum spanning tree was generated using the MSTree V2 algorithm in the stand-alone GrapeTree visualization program.

### 2.5 Resistome analysis

Resistance genes and point mutation content were obtained using two different tools: Amrfinder v.3.1.1b, developed by NCBI selecting *Campylobacter* species parameter (Galaxy-ARIES; Feldgarden et al., 2019), and Staramr v.0.9.1 tool, enabling the scanning for point mutations for *Campylobacter* species (Galaxy Europe; Bharat et al., 2022).

### 2.6 Virulome analysis

Bacterial virulome identification was performed using the Virulence Factor of Pathogenic Bacteria server (VFDB, http://www.mgc.ac.cn/VFs/; Chen et al., 2005, 2012, 2016; Yang et al., 2008; Liu et al., 2018). Additionally, the presence of genes involved in “capsule biosynthesis and transport” and genes categorized as “immune evasion-LOS” was verified using a custom-built database, using *C. jejuni* NCTC 11168 (AL111168.1) as the reference genome. The custom database was built using ABRicate version 1.0.1 (Seemann T, Abricate, GitHub, https://github.com/tseemann/abricate). All genomes were queried against the databases with ABRicate using coverage and identity cutoff of 80%.

The results obtained from VFDB and ABRicate were used to generate a gene presence/absence matrix (Supplementary Table S2). Where two or more copies of the same gene were identified, they were treated as a single hit. The matrix was used to create concatenated sequences for all the samples, which were combined to obtain a binary alignment. A dendrogram was constructed using IQtree version 1.6.9 (Nguyen et al., 2015) using the obtained binary alignment as an input and using default settings. GTR2 + FO + R3 model was selected using ModelFinder feature in IQTree (Kalyaanamoorthy et al., 2017). The tree was annotated with iTOL version 6 (Letunic and Bork, 2007). The genes belonging to the T6SS system present in the genomes were analyzed using the web-based resource Type VI Secretion System Resource (SecReT6) version 3.0. (https://bioinfo-mml.sjtu.edu.cn/SecReT6/t6ss_prediction.php; Supplementary Table S3; Zhang J. et al., 2023).

### 2.7 Plasmid content analysis

The MOB-Recon v.3.0.3 tool predicted sequences’ origin (plasmid or chromosome) (Galaxy Europe; Robertson and Nash, 2018). MOB-Recon differentiated and reconstructed individual plasmid sequences from draft genome assemblies using the plasmid reference databases, detecting circular contigs. The screening was performed with a minimum sequence identity and coverage of 80%. Contigs under 1,000 bp were not considered. Only the plasmids described as circular by this tool were selected and annotated by the Bakta server (http://bakta.computational.bio/, accessed in March 2023; Schwengers et al., 2021).

Plasmids bigger than 10 kb were also subjected to a Pangenome analysis by the Genomic Context View (GView) server (https://server.gview.ca/; Petkau et al., 2010). The BLAST parameters used were *e*-value (<1*e*−10), alignment length cutoff 100%, and identity cutoff value 80%. The pangenome was constructed by iteratively appending unique regions onto an initial seed sequence.

A MAFFT multiple alignment of the plasmid nucleotide sequences, using the default parameters, was performed by the online server MAFFT v. 7 (https://mafft.cbrc.jp/alignment/server/; Katoh and Standley, 2013). This tool performed a sequence alignment using Fast Fourier Transforms. The Neighbor-Joining (NJ) method and the Juke-Cantor model were used to perform a phylogenetic tree by Phylo.io 1.0.0 (Robinson et al., 2016). Only plasmids bigger than 10 kb were selected for MAFFT analysis. These two analyses also included a selection of 11 plasmids, called reference plasmids in this study, based on the MOB and Rep type and the Mash nearest neighbors obtained with the MOB-recon analysis.

## 3 Results

### 3.1 MLST and cgMLST analysis

The WGSs of 133 human non-epidemiologically related *Campylobacter* spp. strains were analyzed and showed a draft genome size between 1.59 and 1.96 Mb with a median N50 of assemblies of 229 kb (IQR = 58–1,000 kb). The number of assembled contigs was between 7 and 99, and the median number of contigs recovered per sample was 32 (Supplementary Table S1). The Kmer-based approach identification and the RefSeq masher confirmed the species of 102 *C. jejuni* and 31 *C. coli* and the absence of contaminated sequences.

Analysis of the 133 *Campylobacter* spp. genomes identified 61 previously described STs and four novel STs (ST-11200, ST-12068, ST-12069, and ST-12070). Seventeen and two different CCs were observed in *C. jejuni* and *C. coli*, respectively. Among *C. jejuni* strains, the main CCs observed were the ST-21 CC, representing 21.24% of the strains, followed by the ST-206 CC (15.53%), the ST-353 CC (9.71%), the ST-354 CC (8.74%), and the ST-658 CC (5.83%). The main STs observed were ST-50, ST-19, and ST-21, inside the ST-21 CC, ST-122 and ST-3335, inside the ST-206 CC and the ST-2116, inside the ST-353 CC. Overall, the predominant CC observed in *C. coli* was the ST-828 CC detected in 80.65% of strains, while only one strain was assigned to ST-1150 CC. The most prevalent *C. coli* STs were ST-7159 and ST-832, members of ST 828 CC (Supplementary Table S1).

A total of 106 cgMLST profiles were observed through *Campylobacter* spp. strains. The most frequent cgMLST types (cgSTs) in *C. jejuni* were the cgST-3020, assigned to six strains and the cgST-11062, assigned to five. In *C. coli,* the most frequent cgSTs were the cgST-35299 (four strains), the cgST-13890 (three strains), and the cgST-20838 (three strains). *Campylobacter jejuni* and *C. coli* did not present common cgMLST. For 19 strains, the exact cgMLST profile was not assigned, as more than one closely matching profile was identified by the Oxford pubMLST (Supplementary Table S1). These strains contained 1–738 mismatches, observing from 99.9 to 80.7% allele match in *C. jejuni* and 98.8 to 45% in *C. coli* (data not shown).

### 3.2 Cluster analysis of human *Campylobacter* spp. strains

**Figure 1:**
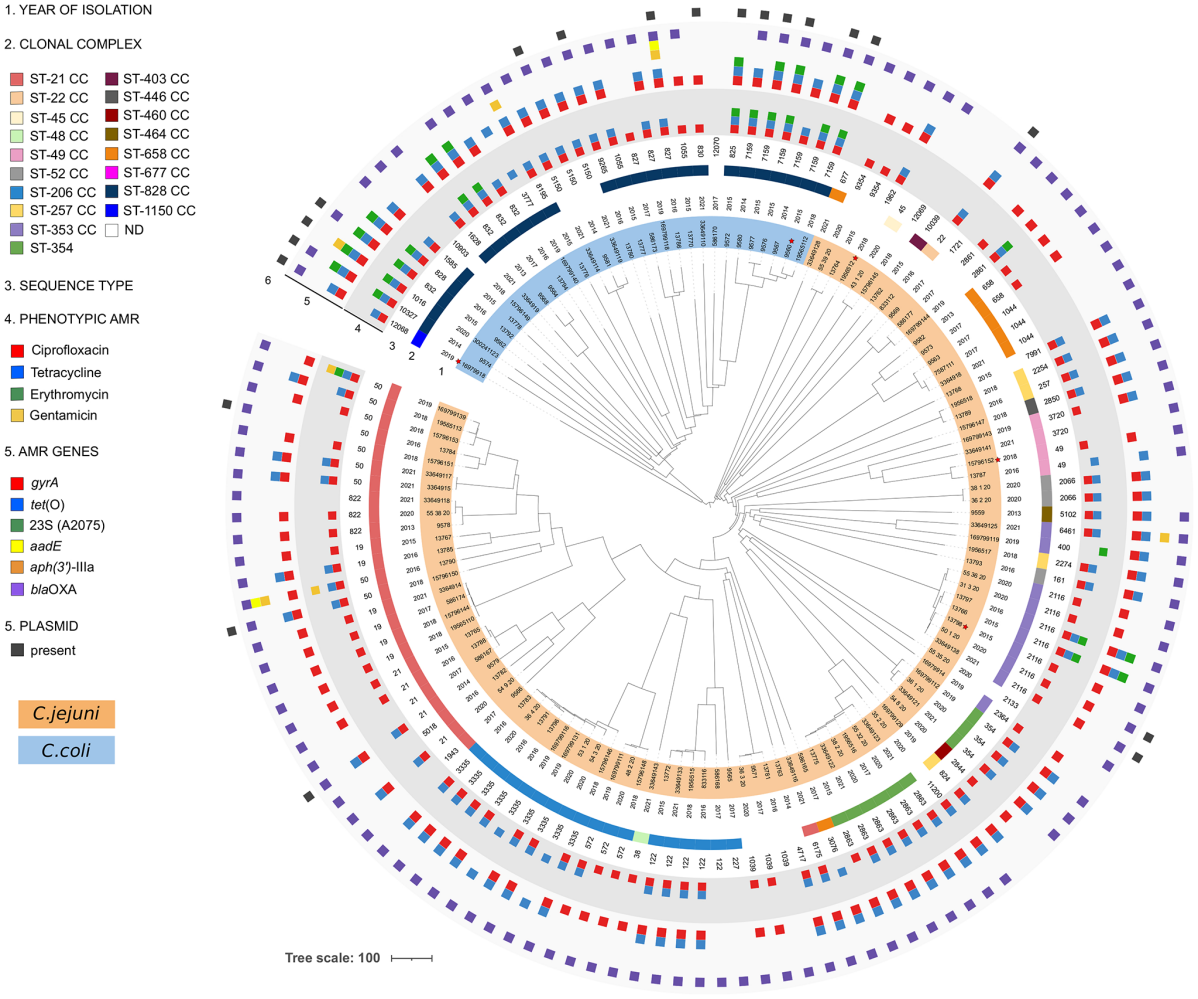
Dendrogram of *Campylobacter jejuni* and *Campylobacter coli* isolated from human samples in Italy between 2013 and 2021. A total of 133 strains were analyzed using cgMLST using a template composed of 1,343 loci. The dendrogram was generated by pairwise comparison of the target core genes and midpoint rooted. Strains marked with red stars were isolated from blood, while the others were isolated from feces. *C. jejuni* and *C. coli* strains are highlighted in orange and blue, respectively.

The dendrogram constructed using cgMLST divided the strains of *C. jejuni* from *C. coli*. Most of the genomes were genetically distant from one another, forming distinct long branches in the tree topology. In particular, the lineage of *C. coli* was split into two branches. The first one contained only one genome assigned to ST-1150 CC, and the second was split further, forming a large clade composed of strains of ST-828 CC or with no CC assigned yet. In general, for *C. coli,* we did not detect clusters of related strains, and only one pair of identical strains was identified within ST-7159 (Figure 1).

For *C. jejuni* strains, instead, we detected several clusters of genomically highly similar strains that included three clusters containing five or more genomes. The biggest one was composed of ST-3335 and contained strains isolated between 2016 and 2020. The pairwise allele distance between genomes in this cluster ranged from one to 35 differing alleles, generally corresponding to the differences in the date or the place of the strain isolation (data not shown). Other two clusters comprised strains assigned to ST-2116 or ST-2863. The allelic differences in the genomes in the ST-2116 cluster ranged between 0 and 23, and for ST-2863, the pairwise distance ranged between zero and 17 alleles. The strains from both clusters were isolated between 2015 and 2021. We also identified several smaller clusters of identical or genetically very similar strains, for instance, within ST-19 or ST-1039. Our dataset’s most populated clonal complex, ST-21 CC, was split between two large clades. The first included ST-21, ST-5018, and ST-1943, and the second comprised ST-19, ST-50, and ST-822. While most strains assigned to this CC were not genetically closely related, two small clusters of similar genomes were noted, one belonging to ST-19 and the other to ST-21 (Figure 1).

### 3.3 Antibiotic susceptibility and genotype analysis

Susceptibility to all antibiotics tested, Cip, E, Gm, and T, was observed in 17.64% (18/102) and 6.45% (2/31) of *C. jejuni* and *C. coli* strains, respectively. All the strains but two (*C. jejuni*) were susceptible to Gm (98.50%). A total of 30.39% (31/102) of *C. jejuni* and 9.68% (3/31) of *C. coli* were only resistant to Cip. A higher number of *C. jejuni* (42.16%; 43/102) and *C. coli* (41.94%; 13/31) were resistant to CipT. 38.71% (12/31) of *C. coli* was characterized by the MDR pattern CipTE; however, a low number (2.94%; 3/102) of CipTE-resistant *C. jejuni* was observed (Supplementary Table S1).

Each interpretation (resistance or susceptible) for an antibiotic susceptibility test result was compared to the presence or absence of a corresponding known resistance gene(s) and/or specific mutations. The *in silico* analysis of the 133 sequenced *Campylobacter* spp. strains revealed the presence of the GyrA (T86I) mutation in 77.45% (79/102) of *C. jejuni* and 83.87% (26/31) of *C. coli*, conferring Cip resistance in all strains except one. The GyrA (T86V) was also present in one Cip-resistant *C. jejuni*, and the double GyrA mutation (T86I, D90N) was present in one Cip-resistant *C. coli*. One *C. jejuni* presented the GyrA mutation (T86A), not conferring Cip resistance. A 20.59% (21/102) *C. jejuni* and a 12.90% (4/31) *C. coli* did not present any GyrA mutation responsible for Cip resistance, being all but one susceptible to this antibiotic (Supplementary Table S1). For the genotype–phenotype correlation, there is no linkage in 1.85% (2/108) of *Campylobacter* strains presenting fluoroquinolone resistance or mutation.

Fifty percent (51/102) of *C. jejuni* and 16.13% (5/31) of *C. coli* did not present any *tet* gene responsible for tetracycline resistance. The *tet*(O) gene was present in 30.39% (31/102) of *C. jejuni* and 67.74% (21/31) of *C. coli*; additionally, 19.60% (20/102) of *C. jejuni* and 12.90% (4/31) of *C. coli* presented the mosaic gene *tet*(O/32/O). The *tet*(W) was present in only one *C. coli* (3.23%; 1/31) (Supplementary Table S1). Two of the *C. jejuni* presenting the *tet*(O) and one of the *C. jejuni* presenting the *tet*(O/32/O) gene were susceptible to T, and four *C. jejuni* resistant to T did not present any *tet* gene. There is no match in the genotype and phenotype correlation in 6.64% (7/81) of *Campylobacter* strains presenting tetracycline resistance or gene.

All but four E-resistant strains presented the 23S RNA mutation A2075G. The transferable *erm*(B) gene was absent. For the genotype and phenotype correlation, there is no match in 2.35% (4/17) of *Campylobacter* strains presenting erythromycin resistance or mutation. Other resistance genes present in this collection were *aadE* [also called *ant(6)-Ia*], *aph(2″)-Ii* (*aadE-Cc*), and *aph(3′)-IIIa* (Supplementary Table S1). Only one of the two strains presenting *aph(3′)-IIIa* gene was resistant to Gm; the other presented a partial *aph(3′)-IIIa*. Instead, one of the two Gm-resistant strains did not present any known mechanism conferring this resistance.

Although the ampicillin resistance was not tested phenotypically in this study, the presence of the *bla*_OXA_ gene and its variants were studied. The *bla*_OXA_ gene was present in a 93.14% (95/102) *C. jejuni* and 80.65% (25/31) *C. coli*. The main *bla*_OXA_ present in *C. jejuni* were *bla*_OXA-193_ 67.65% (69/102) and *bla*_OXA-466_ 7.84% (8/102). *Campylobacter coli* mainly presented *bla*_OXA-193_ 29.03% (9/31) and *bla*_OXA-489_ 19.35% (6/31). A 6.86% (7/102) of *C. jejuni* and a 19.35% (6/31) of *C. coli* did not present *bla*_OXA_ genes (Supplementary Table S1). The 50S rRNA L22 A103V mutation in 15 *C. jejuni* and seven *C. coli* did not confer any resistant pattern to the strains. Considering the four tested antibiotics, only nine strains (6.7%) did not present a correlated pheno-genotype (Supplementary Table S1). The presence of specific antibiotic resistance genes generally corresponded with the cgMLST dendrogram, and strains placed closer together were resistant to the same antibiotics. Some exceptions were noted, however, including two multidrug-resistant strains in the ST-2116 cluster (Figure 1).

### 3.4 Distribution of virulence genes

**Figure 2:**
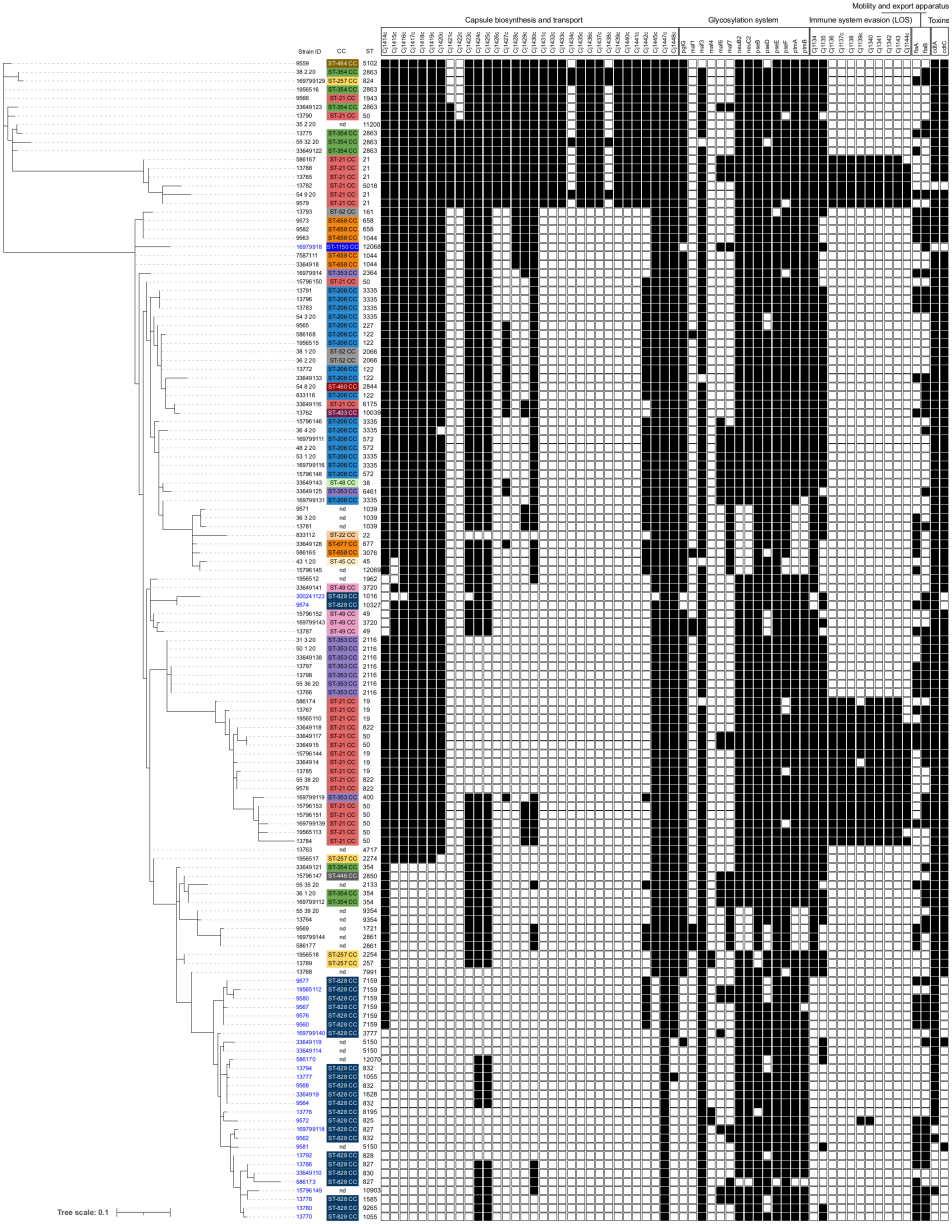
Presence and absence of virulence genes divided by category, in 133 genomes of *Campylobacter jejuni* and *Campylobacter coli* isolated from humans. The dendrogram was constructed using a binary presence/absence matrix of 134 *Campylobacter*-specific virulence genes. Genes detected in >1% and < 99% of genomes are shown (black boxes). IDs are colored according to species: *C. jejuni* are shown in black and *C. coli* in blue. Clonal complexes (CC) are color-coded, and individual Sequence Type (ST) are shown.

The *Campylobacter* spp. collection presented a virulome composed of 158 identified genes out of the 32,827 virulence factor-related genes settled in the virulence finder database VFDB. Out of 158 virulence genes identified, 134 *Campylobacter* spp. specific genes were used to generate a dendrogram based on the binary gene presence/absence matrix. We observed significant differences in the number of virulence genes detected in the individual genomes, with a minimum of 78 genes and a maximum of 125 (Supplementary Tables S2, S3). Fewer virulence genes were found in *C. coli* strains than in *C. jejuni.* The strains with the most detected virulence genes belonged to ST-21 and ST-5018 (ST-21 CC). Of these 134 *Campylobacter* spp. specific genes, 68 virulence genes were conserved in 99% of analyzed strains, and five genes were detected in one strain of *C. jejuni* only. The remaining 61 genes belonged to five separate categories: Capsule biosynthesis and transport (CBT), Glycosylation system, Immune system evasion (lipooligosaccharides—LOS), Motility, and export system and Toxins (Supplementary Table S2; Figure 2). The most significant difference between the strains was observed in the presence or absence of CBT and LOS genes.

The dendrogram grouped the dataset into specific clusters based on the similarity of virulomes, and the individual clusters generally corresponded to *Campylobacter* species and specific CCs, but with some exceptions (Figure 2). ST-21 CC was divided into two clusters, the first grouping ST-21 and ST-5018 and the second composed mainly of ST-50 and ST-19 strains. Strains from both clusters differed from the others in the dataset by the presence of nine genes in the LOS cassette (*Cj1136–Cj1144c*; Figure 2); however, the first cluster contained additional nine genes in the CBT cassette. Moreover, while most *C. coli* genomes were grouped, three strains were found among *C. jejuni* strains, suggesting a possible gene exchange between the two species.

The VFDB analysis showed that 75 out of 133 strains (48 *C. jejuni* and 27 *C. coli*) carried virulence genes associated with bacterial species different from *Campylobacter* spp. (Supplementary Table S3). Thirty *C. jejuni* and two *C. coli* strains carried three virulence genes associated with ACE Type VI secretion system (T6SS) of *Escherichia coli*, T6SS of *Aeromonas* spp., and virulence-associated secretion (VAS) effector protein of *Vibrio* spp. Six of these 30 *C. jejuni* strains belonged to ST-354 CC and nine to ST-353 CC (Supplementary Table S3). Twenty out of 133 strains (9 *C. jejuni* and 11 *C. coli*) carried a virulence gene linked to the Lvh type IVA secretion system (*Legionella* spp. *vir* homologs). Among the *C. jejuni* strains, six were classified as ST-353 CC. Likewise, among the *C. coli* strains, nine were classified as ST-828 CC. Twenty-three *Campylobacter* spp. strains (12 *C. jejuni* and 11 *C. coli*) showed a gene coding for Phytotoxin phaseolotoxin (*Pseudomonas* spp.), and no clear correlation with any CCs was observed (Supplementary Table S3). Eight out of the 31 strains of *C. coli* belonging to ST-828 CC presented virulence genes associated with serum resistance and immune evasion, anti-phagocytosis, and other functions classified by the VFDB tool as lipopolysaccharide (LPS) gene of *Francisella* spp., capsule of *Klebsiella* spp., and O-antigen of *Yersinia* spp., respectively (Supplementary Table S3).

### 3.5 Plasmid analysis

MOB-recon analysis classified the contigs of all the strains in chromosomal or plasmidic, assigning plasmidic contigs as circular or incomplete (Supplementary Table S4). This tool identified plasmid-derived contigs in all 102 *C. jejuni* strains and only 18 *C. coli* (Supplementary Table S4). Additionally, 19 out of 133 *Campylobacter* spp. strains presented one plasmid classified as circular, while one strain (833,112 strain) presented three circularized plasmids (Table 1; Supplementary Table S1). Six *C. jejuni* and 14 *C. coli* presented circularized plasmids (Table 1). All circularized plasmids exhibited homology with previously characterized *Campylobacter* spp. plasmids (Table 1). Plasmid sizes varied between 2,427 and 49,853 bp (Table 1). Four circularized plasmids <10 kb were defined as cryptic. The distribution of strains containing circularized plasmids was observed across the cgMLST dendrogram except for *C. coli* ST-7159 strains. Five out of six ST-7159 strains contained a plasmid (Figure 1). Four out of the 20 plasmids presented the *tet*(O) gene (Table 1).

**Table 1:** MOB-recon *Campylobacter* spp. circularized plasmids results and antimicrobial resistance genes.

Plasmid name	Plasmid acc. n.	Strain	Resistance gene	Plasmid size (bp)	GC%	CDS^a^	*rep* type	*rep* acc. n.^b^	Relaxase type	Relaxase acc. n.^b^	Mash nearest neighbor^b^
**p9574**	OQ553938	*C. coli* 9574	-	2,427	26	3	-	-	-	-	CP007185
**p300241123**	OQ553956	*C. coli* 300241123	-	2,908	30	4	rep_cluster_840	NC_004997	-	-	AY256846
**p9562**	OQ553936	*C. coli* 9562	-	3,267	32	4	rep_cluster_950	CP017855	-	-	MH634988
**p833112_4kb**	OQ553947	*C. jejuni* 833112	-	4,367	31	7	rep_cluster_795	NC_008052	MOBP	NC_008051	MH634989
**p9581**	OQ553943	*C. coli* 9581	-	24,869	29	30	-	-	MOBP	CP017870	**CP017870**
**p9576**	OQ553939	*C. coli* 9576	-	25,341	29	31	-	-	MOBP	CP017870	CP017870
**p9577**	OQ553940	*C. coli* 9577	-	25,341	29	31	-	-	MOBP	CP017870	CP017870
**p833112_26kb**	OQ553949	*C. jejuni* 833112	-	26,724	29	45	-	-	MOBP	CP006703	**CP017231**
**p19565112**	OQ553953	*C. coli* 19565112	-	27,225	29	35	-	-	MOBP	CP006703	CP006703
**p9560**	OQ553935	*C. coli* 9560	-	27,235	29	36	-	-	MOBP	CP006703	CP006703
**p33649110**	OQ553954	*C. coli* 33649110	-	27,538	30	33	-	-	MOBP	CP006703	**CP045792**
**p9580**	OQ553942	*C. coli* 9580	-	27,544	30	33	-	-	MOBP	CP006703	CP045792
**p16979918**	OQ553952	*C. coli* 16979918	-	30,346	28	38	-	-	MOBP	CP006703	CP017231
**p50_1_20**	OQ553934	*C. jejuni* 50_1_20	-	30,346	28	38	-	-	MOBP	CP006703	CP017231
**p33649138**	OQ553955	*C. jejuni* 33649138	-	30,348	28	38	-	-	MOBP	CP006703	CP017231
**p13780**	OQ553944	*C. coli* 13780	-	30,631	29	39	-	-	MOBP	CP006703	**CP006703**
**p13784**	OQ553945	*C. coli* 13784	-	35,326	26	47	rep_cluster_1502	**CP013734**	MOBP	**NZ_AZNS01000034**	CP014746
**p9572**	OQ553937	*C. coli* 9572	-	39,389	28	45	-	-	MOBP	**NC_022355**	**CP043764**
**p833112_43kb**	OQ722348	*C. jejuni* 833112	*tet*(O)	43,681	28	47	-	-	MOBP	NC_022355	**CP022471**
**p15796150**	OQ553951	*C. jejuni* 15796150	*tet*(O)	44,310	28	50	-	-	MOBP	NC_022355	CP022471
**p9579**	OQ553941	*C. jejuni* 9579	*tet*(O)	44,469	30	49	rep_cluster_475	**KX686749**	MOBP	NC_022355	**CP011017**
**p13786**	OQ553946	*C. coli* 13786	*tet*(O)*, aph(2″)-Ii, aph(3′)-IIIa*Δ	49,853	29	57	-	-	MOBP	NC_022355	CP043764

^a^Bakta v.1.7.0 predicted reading frames; ^b^In bold, reference plasmids selected for the Gview and MAFFT_NJ plasmid analysis; Δ: partial gene.

MOB-recon analysis confirmed the presence of replicase (*rep*) and *mob* relaxase genes. Rep proteins were present in three circularized cryptic plasmids, presenting high homologies to three previously described cryptic plasmids (Table 1). The Bakta server identified a replication initiation protein Rep_3 (RepB family) in the fourth cryptic p9574; this protein was misidentified by the MOB-recon tool.

In 11 strains without circularized plasmids, MOB-recon identified 12 non-circularized plasmid contigs that encoded MOB relaxases (and one also encoded a Rep protein). Four non-circularized plasmid contigs also presented the *tet*(O) gene (Supplementary Tables S1, S4).

**Figure 3:**
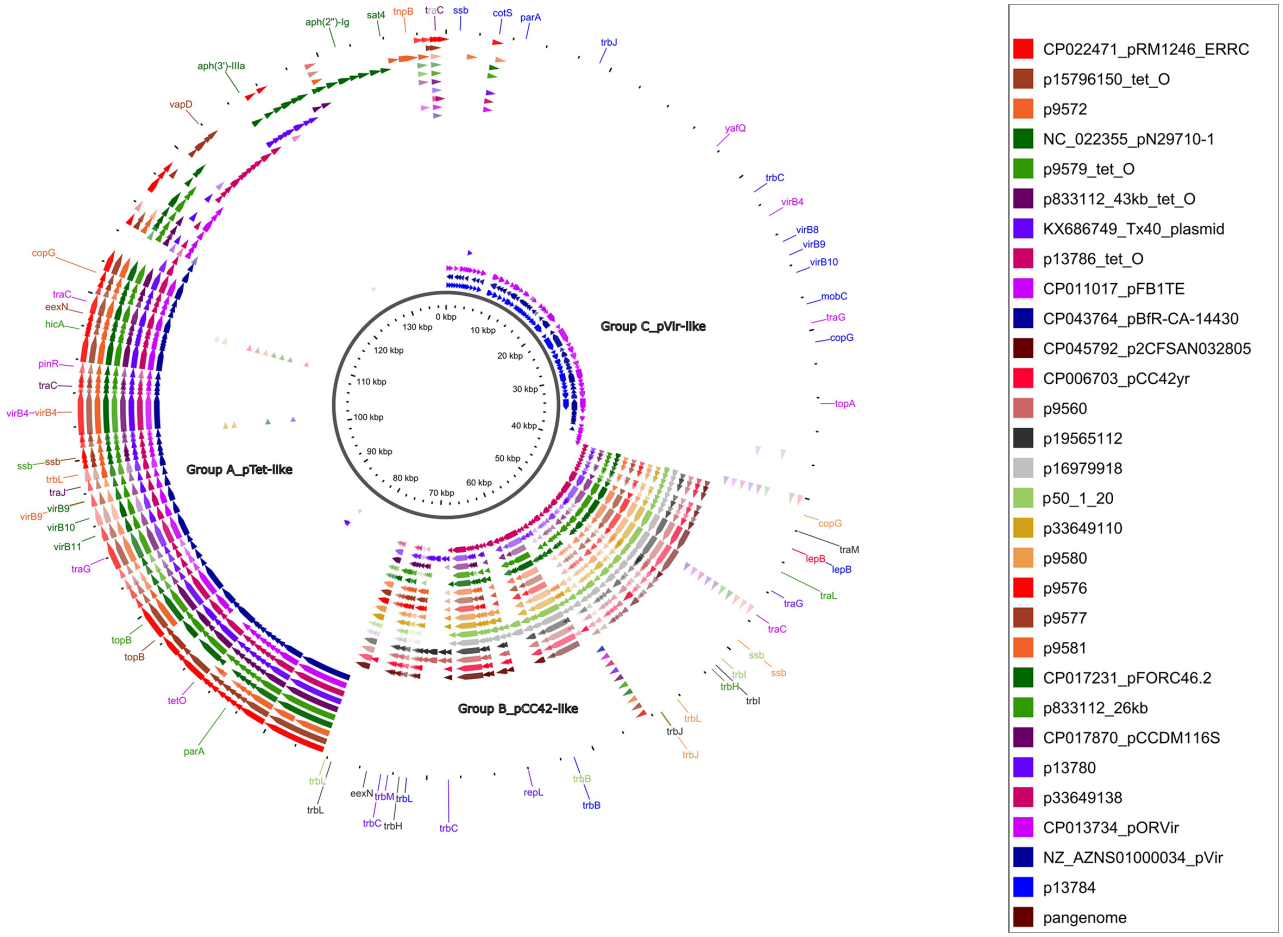
Pangenome analysis of circularized *Campylobacter* spp. plasmids bigger than 10 kb. GView server showed a circular figure with 18 circularized plasmid sequences plus 11 reference plasmids. Individual colored slots (circles) in the figure represent one plasmid sequence. Circles showed regions with BLAST hits between the constructed pangenome and the other uploaded sequences. The significant gaps showed regions missing from the pangenome but found in one of the other sequences. The dark brown circle showed the constructed pangenome using all the uploaded concatenated plasmid sequences. Individual arrows represent the CDS.

**Figure 4:**
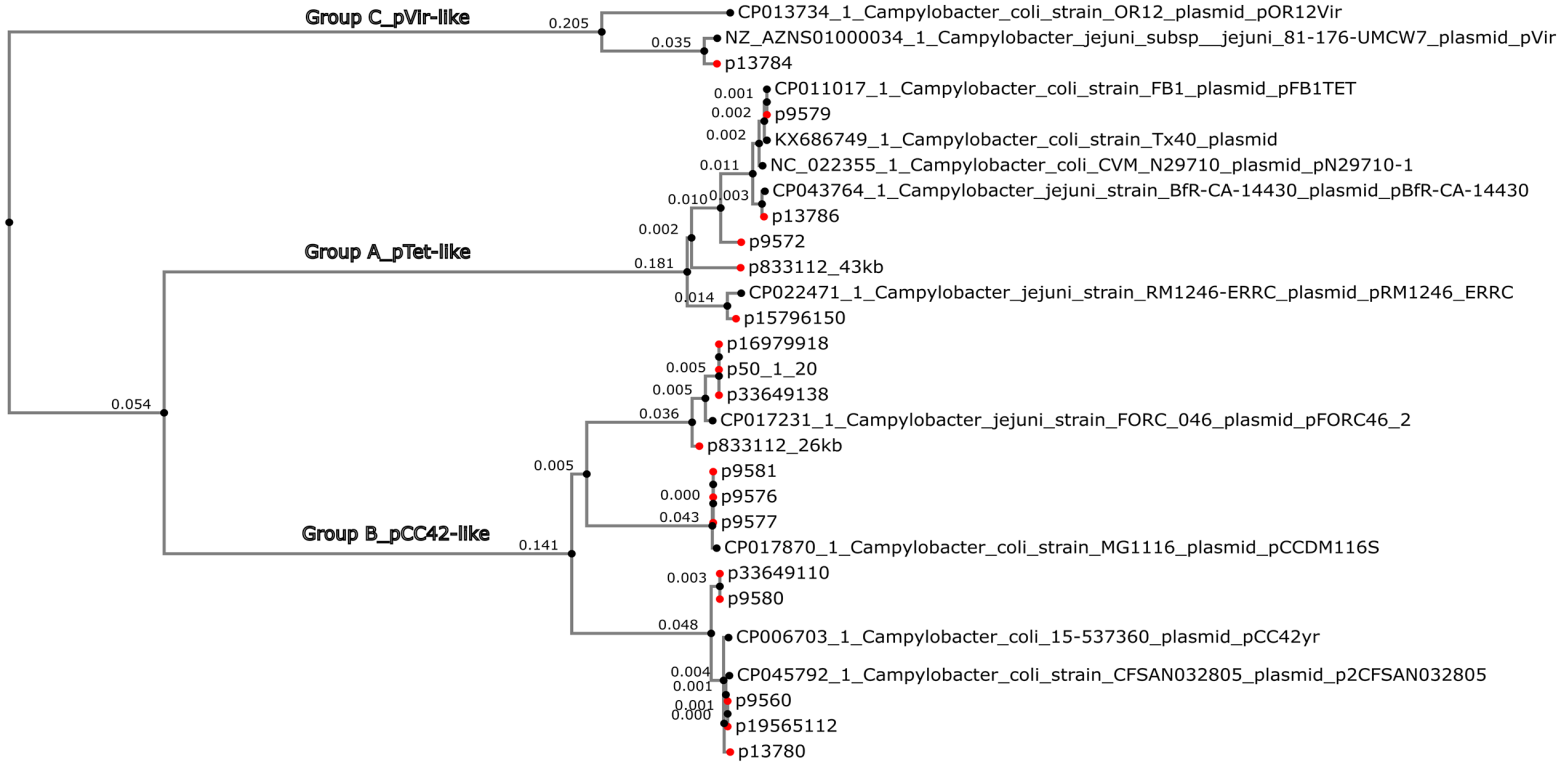
Phylogeny of *Campylobacter jejuni* and *Campylobacter coli* circularized plasmids bigger than 10 kb. A MAFFT multiple alignment of the plasmid nucleotides was performed. The Neighbor-Joining method and Juke-Cantor model were used to perform a phylogenetic tree by Phylo.io 1.0.0. Eighteen circularized plasmids (red dots) plus 11 reference plasmids are represented.

The GView pangenome analysis, of 18 circularized plasmid sequences larger than 10 kb and 11 reference plasmids added for comparative purposes (Table 1; Figure 3), revealed a comprehensive overview through a circular map displaying the pangenome BLAST atlas of all sequences (Figure 3). The GView analysis grouped the plasmids into three main groups (A–C). This grouping was further supported by the NJ-phylogenetic tree generated through MAFFT analysis (Figure 4), reinforcing the consistency of the results obtained from both methodologies. Within these groups, distinct clades of pTet-like, pCC42-like, and pVir-like plasmids emerged, aligning with their respective reference plasmids.

The *tet*(O)-carrying plasmids plus the p9572 were classified into group A as pTet-like plasmids, alongside the five reference plasmids previously described as pTet (Table 1; Figure 3). The 39 kb-p9572 did not present the *tet*(O) gene but maintained most of the pTet-plasmid backbone. This strain presented the *tet*(O) gene in a 173 kb-contig classified as chromosomal by MOB-recon (Supplementary Table S4) and presented a 97% of coverage and 99.91% of identity with the *C. coli* strain meC0467 chromosome (CP027638). The p13786 classified as group A (pTet-like) presented a partial *aph(3′)-IIIa* gene, intact in the Tx40 plasmid and pN29710-1 reference plasmids. p1378 also presented the *aph(2″)-Ii* gene. These four pTet-like plasmids were observed in one ST-828 CC *C. coli* and three *C. jejuni* of ST-21 CC and ST-22 CC (Supplementary Table S1).

Group B, or pCC42-like, was the biggest one (70.58% of the total of circularized plasmids), with 17 plasmids ranging from 25 to 30 kb (four reference plasmids and 12 circularized plasmids); their plasmid backbone was mainly constituted by a significant coding region for the Tra/Vir type IV secretion system (T4SS). Seventy-five per cent (9/12) of these plasmids were associated with *C. coli.* Among these *C. coli*-associated plasmids, a subset of 55.5% (5/9) was identified in ST-7159 (ST-828 CC) strains isolated in different years; nevertheless, plasmids grouped in two closely related subclades (Figure 4). The remaining plasmids, which formed a third sub-clade, were found in three strains of *C. jejuni* and one strain of *C. coli*. Interestingly, the *C. coli* strain identified in this sub-clade did not belong to the ST-828 CC but was classified as ST-12068 (ST-1150 CC).

Group C, or pVir-like, was constituted by the p13784 and two reference plasmids, pOR12Vir and pVir, previously described as *Campylobacter* spp. virulence plasmids (Figure 3). The pVir-like p13874 presented 97% of coverage and 97.99% of identity with pVir and 95% of coverage and 97.84% of identity with pOR12Vir. The plasmids of this group were characterized to present seven genes encoding homologs of type IV secretion proteins and are clustered in a region spanning 8.9 kb. One of these genes was previously identified by VFDB analysis as Lvh type IVA secretion system (*Legionella* spp. *vir* homologs).

## 4 Discussion

Campylobacteriosis cases have increased worldwide (Whiley et al., 2013; Gibbons et al., 2014; EFSA and ECDC, 2021, 2022). Nevertheless, this infection is frequently underdiagnosed and underreported (Gibbons et al., 2014). Genetic characterization and comparison of *Campylobacter* spp. play a crucial role in advancing our knowledge of the dynamics and characteristics of circulating lineage clusters. A high genetic diversity of *C. jejuni* strains has been extensively documented worldwide and possesses a notable capacity for genetic exchange, enhancing its adaptability (Golz et al., 2020; Bravo et al., 2021; Harrison et al., 2021; Ocejo et al., 2021; Prendergast et al., 2022; Zhong et al., 2022; Hull et al., 2023). Notably, extensive introgression (transfer of genetic material between different species), predominantly from *C. jejuni* to *C. coli,* has been observed, resulting in the replacement of approximately 10 and 23% of the *C. coli* core genome with *C. jejuni* DNA in ST-828 and ST-1150 CCs, respectively (Sheppard et al., 2008; Tanoeiro et al., 2022).

In this study, genome analysis showed a significant genetic divergence in the selected collection. The assignment to different STs and CCs demonstrated that 41% of the *C. jejuni* strains were grouped into two main CCs, ST-21 CC and ST-206 CC, defined as host generalists (capable of infecting wide range of host species) (Baumler and Fang, 2013; Thépault et al., 2017; Bravo et al., 2021; Mouftah et al., 2021; Dessouky et al., 2022; Ghielmetti et al., 2023).

Our results agree with several studies, demonstrating that ST-21 CC is prevalent in the *C. jejuni* population in humans in Italy (Manfreda et al., 2016; Di Giannatale et al., 2019; Ocejo et al., 2021; Conesa et al., 2022; Iannino et al., 2022). ST-21 CC is also among the most reported CC in poultry and is often associated with cattle, wild birds, sheep, dogs, dairy products, and water (Kwan et al., 2008; Cuevas-Ferrando et al., 2020; Conesa et al., 2022; Deblais et al., 2023). ST-206 CC has also been found frequently in humans in Germany, chicken meat, dogs in Italy, chickens in Poland, and sheep in Spain (Rosner et al., 2017; Wieczorek et al., 2017; Di Giannatale et al., 2019; Ocejo et al., 2021; Iannino et al., 2022). ST-353 CC, detected in 9.8% (10/102) of the strains, has been classified as a chicken specialist due to its prevalence in this host (Cobo-Díaz et al., 2021). *Campylobacter coli* showed a low CC heterogeneity, as all the strains except one were classified as ST-828 (Mouftah et al., 2021).

Many studies have suggested approaches to overcome the limited discriminatory power of the conventional seven loci MLST by exploiting WGS data (Cody et al., 2017). Our collection presented a high variability of cgMLST profiles, aligning with our objective of choosing non-epidemiologically linked strains for this study. The cgMLST dendrogram highlighted that most strains analyzed were not genetically related. This was particularly evident for *C. coli*, where clusters of similar genomes remained undetected. The high variability of cgSTs circulating in our country suggests that human infections in Italy are usually sporadic, likely associated with improper handling of food products, which mirrors the epidemiology of campylobacteriosis in EU (EFSA and ECDC, 2022; Liu et al., 2022).

Only a few small groups of strains were classified as the same cgST, mainly associated with the ST-2116, ST-2863, and ST-3335. These strains were isolated in different years, suggesting they did not originate from an outbreak but were potentially acquired from a common source. Recent studies have indicated that ST-2116 and ST-2863 are the most prevalent STs isolated from broilers in Italy, exhibiting genetic homogeneity (Di Giannatale et al., 2019; Marotta et al., 2023). This suggests that human infections caused by these clones, which are extensively circulating in Italy, are likely linked to broilers and the consumption of contaminated poultry meat and related food products. Conversely, infections involving ST-3335 have been attributed to cattle and sheep, indicating a probable connection with ruminant reservoirs (Arning et al., 2021). Therefore, it is crucial to conduct further research utilizing more precise attribution models to examine the sources of infection.

The analysis of the *Campylobacter* spp. collection revealed a diverse virulome among individual genomes. Notably, *C. jejuni* strains carried more virulence genes than *C. coli* strains (Bravo et al., 2021; Hull et al., 2023). The virulome of *Campylobacter* spp. has mainly been studied in *C. jejuni*, and most of the commonly used virulence factor databases have been constructed based on the reference genome of this species. The low number of virulence factors detected in *C. coli* may be attributed to either the absence of *C. coli*-specific genes in the database queried or a sequence identity below the VFDB tool’s threshold between the *C. coli* virulence genes and those in the *C. jejuni* reference genomes.

The dendrogram, generated from a binary matrix indicating the presence or absence of virulence genes, provided insights into the clustering of strains based on their virulomes. The identified clusters corresponded to specific *Campylobacter* species and CCs. Specific virulence genes were associated with particular STs, such as ST-21 and ST-5018 (ST-21 CC), highlighting the potential role of these genes on the pathogenicity of *Campylobacter* spp. (Zhang D. et al., 2023). However, few exceptions were observed, including two distinct clusters within the ST-21 CC, suggesting genetic differences within this complex. A nine-gene LOS cassette also distinguished the strains in these clusters, with one cluster carrying additional genes in the CBT cassette. We did not detect any significant difference between the strains isolated from blood and fecal samples, which could suggest that the cases of bacteraemia were not associated with the changes in the strain’s virulence but rather with the host-specific factors. Interestingly, the presence of *C. coli* strains among *C. jejuni* strains suggested the possibility of gene exchange between these two species.

A recent study on the genomic diversity and virulence profile of the genus *Campylobacter* demonstrated that the number of genes has undergone expansion or contraction during the evolution of different *Campylobacter* species (Zhong et al., 2022). VFDB analysis revealed that many strains (75 out of 131) carried virulence genes associated with bacterial species other than *Campylobacter* spp., suggesting potential gene transfer events between different bacterial species. *Campylobacter jejuni* strains, particularly those belonging to ST-354 CC and ST-353 CC, both ranking among the top four most frequent STs in our collection, carried virulence genes associated with *E. coli*, *Aeromonas* spp., and *Vibrio* spp. This highlights a wide variety of virulence factors within specific clonal complexes. This study suggests that the T6SS virulence genes of *E. coli*, and *Aeromonas* spp. are probably acquired from other bacterial species upon multiple and independent genetic uptakes. The identification of these virulence genes in two *C. coli* strains suggests a more comprehensive bioinformatic analysis of T6SS and the determination of associated effectors in *C. coli* is still needed.

The presence of virulence genes associated with other species in *C. coli*, such as LPS gene of *Francisella* spp., capsule of *Klebsiella* spp., and O-antigen of *Yersinia* spp., underscores their importance for the bacterium’s survival during colonization and infection of host tissues (Lehmann et al., 2006). It has been demonstrated that *C. jejuni* bind to a diverse range of host glycans that are potentially crucial for the initial attachment to and continued colonization of the host (Morrow et al., 2005; Day et al., 2009); a better comprehension of the factors involved in glycan expression and recognition by *C. coli* and the host may elucidate the mechanisms involved in *C. coli* commensal colonization and pathogenic infection.

Antibiotic resistance surveillance in *Campylobacter* spp. has become a challenge in recent years. Our study showed high levels of resistance to Cip and T and low levels of resistance to macrolides and aminoglycosides, as described previously (EFSA and ECDC, 2022). Despite a generally high concordance between antibiotic resistance genotypes and phenotypes, tetracycline exhibited the lowest correlation between the presence of the *tet* gene and susceptibility patterns. The absence of complete matching is widely described worldwide due to a misdetection of some AMR mechanisms (Engberg et al., 2001; Luo et al., 2003; Painset et al., 2020; Rokney et al., 2020; Mouftah et al., 2021; Dessouky et al., 2022). Without a precise mechanism, erythromycin and/or tetracycline resistance could be present due to mutations in CmeABC operon, its transcriptional regulator CmeR or in its promoter (Aksomaitiene et al., 2018; García-Fernández et al., 2018; Oncel et al., 2023). CmeABC efflux pump can also synergise with the GyrA and 23S rRNA mutations in maintaining high levels of resistance to fluoroquinolones and macrolides (Payot et al., 2006). Using an automated annotation pipeline to detect antibiotic resistance mechanisms could mislead some mechanisms. The presence or absence of efflux pumps, membrane permeability, frameshift mutations, and the existence of mosaic or new resistance genes could impact the phenotypic resistance pattern (Hormeño et al., 2020). Technical issues such as poor-quality sequences, assembly errors, and incorrect analysis may also contribute to discrepancies in antibiotic resistance patterns (Bortolaia et al., 2020; Elhadidy et al., 2020). Addressing these complexities is crucial for accurate and comprehensive antibiotic resistance surveillance in *Campylobacter* spp.

Consistent with global observations, this study identified the GyrA (T86I) mutation and the *tet*(O) gene as the primary mechanisms responsible for quinolone and tetracycline resistance, respectively (Luo et al., 2003; García-Fernández et al., 2018; Ocejo et al., 2021). A lack of a clear association between increased virulence genes and antibiotic resistance profiles in *C. jejuni* or *C. coli* was observed. *Campylobacter coli*, despite having fewer virulence genes, exhibited higher resistance, including MDR. The contrasting situation with *C. jejuni* ST-21 CC, being the most virulent CC and presenting a high percentage of strains resistant to CipT. Moreover, susceptible strains were distributed along the presence/absence virulence gene dendrogram without a clear correlation, demonstrating that the susceptibility of *Campylobacter* spp. strains may not be directly linked with the degree of virulence the strains exhibit.

The presence of plasmids in *Campylobacter* spp. collections isolated from humans have not been extensively studied, and few studies have been conducted on this topic (Lee et al., 1994). Nevertheless, with the increasingly widespread adoption of WGS, recent studies have revealed a notable occurrence of plasmids in *Campylobacter* spp. strains (Marasini and Fakhr, 2017; Deblais et al., 2023; Ghielmetti et al., 2023; Hull et al., 2023). Fifteen percent of strains of our collection presented plasmids; nevertheless, many linear contigs were classified by the MOB-recon tool as plasmidic, suggesting a potentially higher prevalence of plasmids beyond those directly identified as circular (Hull et al., 2023).

The *tet*(O) genes were mainly found on the bacterial chromosome, with only a small percentage found in circularized plasmids, aligning with previous findings (Ghielmetti et al., 2023). One of the plasmids carrying *tet*(O), the pTet-like p13786, also showed the *aph(3′)-IIIa* and a partial *aph(2″)-Ii* genes, which did not confer resistance to Gm. The presence of the pTet family in *C. jejuni*, containing multiple resistance genes, suggested a potential role in enhancing multidrug resistance, promoting bacterial survival, and contributing to virulence. These plasmids also presented genes coding type II toxin-antitoxin system HicA-HicB, the CagA pathogenicity island protein, the VapD virulence-associated protein and several genes encoding predicted type IV secretion/conjugal transfer proteins with homology to T4SS. These findings suggested a complex interplay between plasmids and the expression of virulence and resistance factors in *Campylobacter* spp. (Batchelor et al., 2004; Wallden et al., 2010; Bunduruș et al., 2023; Gabbert et al., 2023; Morita et al., 2023) The p13876, next to the *tet*(O) gene, presented two Type II CRISPR-associated endonucleases coding genes, *cas1* and *cas2,* described in the *Campylobacter* spp. chromosome rather than in plasmids. Together with CRISPR, these proteins could provide acquired genetic immunity against the entry of mobile genetic elements after infection by a phage expressing a Cas4-like protein (Hooton and Connerton, 2014; van Vliet et al., 2021). This CRISPR-mediated autoimmunity could, therefore, profoundly direct the shape of evolution in these species (Lee et al., 1994; Marasini and Fakhr, 2017; Deblais et al., 2023).

Cryptic plasmids have been identified in several *Campylobacter* species encoding for replication proteins (Alfredson and Korolik, 2003; Hiett et al., 2013). The cryptic plasmids found in our study presented genes encoding for replication proteins and shared homology with previously described cryptic plasmids deposited in the GenBank database. The precise role of these small plasmids remains unclear. Some studies suggest that those may act as modifiable vectors for genetic innovation in other species, such as *Aeromonas salmonicida* (Attéré et al., 2017).

Gview analysis and the Phylogenetic NJ-tree classified plasmids found in this study into three groups or clades (A–C), preserving well-determined lineages. These phylogenetic tree clades supported the categorization obtained with the pangenomic analysis, revealing sub-braches indicative of evolutionary divergence within each clade.

Group A comprised pTet-like plasmids, which presented essential genes responsible for conjugation, virulence (including the Type IV secretion system), and were associated with carrying MDR genes (Bacon et al., 2002; Marasini and Fakhr, 2016; Hull et al., 2023; Morita et al., 2023).

Group B, previously known as pCC42-like plasmids, was formed by plasmids coding for conjugative transfer genes (*trb*/*tra*) with type IV secretion system genes (*virB3/B4*) (Bacon et al., 2002; van Vliet et al., 2021; Ghielmetti et al., 2023). This plasmid class was prevalently associated with ST-7159 *C. coli* strains isolated in different years, emphasizing the persistence of this plasmid-associated sequence type over time.

Group C, or pVir-like, included p13784, presenting significant homology with pVir. It encoded genes homologous to the type IV secretion system in *Helicobacter pylori*. This system was involved in invading *C. jejuni* strain 81–176, which demonstrated virulence in human volunteer studies (Bacon et al., 2002; Ghielmetti et al., 2023).

It is noteworthy that pVir-like plasmids are less prevalent than pTet-like plasmids in *Campylobacter* spp. strains (Marasini et al., 2018). A previous study conducted with *Campylobacter* spp. strains isolated from retail meat corroborated our classification groups, including an additional fourth group of plasmids <10 kb called in our study as cryptic plasmids (Marasini et al., 2018).

This genomic approach provides an interesting understanding of plasmid diversity, facilitating the identification of distinct groups and highlighting the evolutionary relationships among the plasmids, thereby contributing to our comprehension of *Campylobacter* spp. plasmid dynamics (Marasini et al., 2018). A more in-depth exploration of the plasmids present in our *Campylobacter* spp. collection, using long-read sequencing technologies, could substantially contribute to providing a more accurate assessment of the proper frequency of plasmid presence within our *Campylobacter* spp. collection.

Our study demonstrated a large genomic diversity within a population of *Campylobacter* spp. associated with human infections in Italy. While some of the detected MLST clusters were previously linked to the consumption or handling of poultry meat, others seem to be associated with different animal sources, including cattle and sheep or domestic pets.

Therefore, it is important to implement surveillance and control measures for *Campylobacter* spp. in broiler farms and dairy production to reduce the carriage of *Campylobacter* spp. and, consequently, decrease the risk of carcass and food contamination during the primary phases of the production chain. Moreover, good safety and hygiene practices must be applied when handling raw meat and, importantly, when preparing artisanal dairy and food products based on unpasteurised milk (Dai et al., 2020; Emanowicz et al., 2021; Al Hakeem et al., 2022).

In addition, considering the consistently high level of antibiotic resistance to fluoroquinolones and tetracycline in Italian strains of *C. jejuni* and *C. coli*, continuous effort must be maintained in limiting the use of antibiotics in the food-producing animals to avoid further spread of the resistant strains (Mouftah et al., 2021; EFSA and ECDC, 2023; Fonseca et al., 2023).

Whole genome sequencing emerges as a valuable tool for developing new strategies to address antibiotic resistance. Accurate whole-genome characterization, and the prediction of antibiotic resistance improves current surveillance programs’ accuracy and effectiveness (Ocejo et al., 2021; Ghielmetti et al., 2023). WGS also gives high resolution typing methods allowing precise differentiation among *Campylobacter* strains. This approach not only facilitates comprehensive “One Health” epidemiological investigations, but also enables the thorough examination of foodborne disease outbreaks related to *Campylobacter* spp. and the tracking of transmission sources (Cody et al., 2019; Zhang D. et al., 2023). The accurate understanding of the source of *Campylobacter*, whether it originates from specific food, environmental reservoirs, or specific animal hosts, is crucial for target intervention and preventive measures.

## Data availability statement

Genome sequences were deposited in GenBank (https://www.ncbi.nlm.nih.gov) under BioProject ID PRJNA913772. Strains were stored under the consecutive BioSample accession numbers: SAMN32306040–SAMN32306055 and SAMN32306053–SAMN32306173. Plasmids were submitted and consecutively assigned to NCBI accession numbers from OQ553934–OQ553947, OQ553949, OQ553951–OQ553956, and OQ722348 (Table 1). Strain-specific details for bacteria can be found in Table 1 and Supplementary Table S1. The presence of genes involved in "capsule biosynthesis and transport" and genes categorized as "immune evasion-LOS" was verified using a custom-built database, using *C. jejuni* NCTC 11168 (AL111168.1) as the reference genome. The custom database was built using ABRicate version 1.0.1 (Seemann T, Abricate, GitHub https://github.com/tseemann/abricate). The results obtained from VFDB and ABRicate were used to generate a gene presence/absence matrix (Supplementary Table S2). The authors confirm all supporting data, code and protocols have been provided within the article or through Supplementary material.

## Author contributions

AG-F: Conceptualization, Data curation, Formal analysis, Investigation, Methodology, Project administration, Software, Supervision, Validation, Visualization, Writing – original draft, Writing – review & editing. AJ: Software, Writing – original draft, Writing – review & editing, Data curation, Validation. FM: Methodology, Writing – review & editing, Data curation. MN: Data curation, Writing – original draft, Writing – review & editing, Methodology. SA: Methodology, Writing – review & editing. SP: Data curation, Methodology, Writing – review & editing. MP: Data curation, Methodology, Writing – review & editing. RR: Methodology, Writing – review & editing. FT: Methodology, Writing – review & editing. GG: Conceptualization, Data curation, Supervision, Writing – review & editing. LV: Conceptualization, Funding acquisition, Investigation, Project administration, Resources, Supervision, Writing – original draft, Writing – review & editing.

## Figures and Tables

**Table:** 

MLST	Multilocus sequence typing
CCs	Clonal complexes
EU	European Union
MSs	Member States
ECDC	European Center for Disease Prevention and Control
STs	Sequence types
cgMLST	Core genome multilocus sequence typing
cgST	cgMLST type
ISS	Istituto Superiore di Sanità
mCCD Agar	Modified charcoal cefoperazone deoxycholate agar
EUCAST	European Committee on Antimicrobial Susceptibility Testing
Cip	Ciprofloxacin
T	Tetracycline
E	Erythromycin
Gm	Gentamicin
ECOFFs	Epidemiological cutoff values
WGS	Whole genome sequencing
CGE	Center for genomic epidemiology
VFDB	Virulence factor of pathogenic bacteria server
GView	Genomic Context View
NJ	Neighbor-joining
CBT	Capsule biosynthesis and transport
LOS	Lipooligosaccharides
T6SS	Type VI secretion system
VAS	Virulence-associated secretion
LPS	Lipopolysaccharide
T4SS	Type IV secretion system

## Footnotes

^1^http://www.eucast.org/clinical_breakpoints/v.11.0

^2^www.eucast.org

^3^https://pubmlst.org/organisms/campylobacter-jejunicoli/
