# Supplementary material for: Antibiotic resistance, plasmids, and virulence-associated markers in human strains of Campylobacter jejuni and Campylobacter coli isolated in Italy
Source: Front Microbiol. 2024 Jan 8;14:1293666. doi: 10.3389/fmicb.2023.1293666 (PMC10800408; doi:10.3389/fmicb.2023.1293666)
Supplement: Supplementary file 1 [file Table_1.DOCX]

Table S1. Summary of genomic features of *Campylobacter* spp. strains sequenced in this study.

| Isolate | Specie | Sample | Year | Genome bp (starAMR) | N50  (starAMR) | n°contigs> 300bp | Closest cgST  (pubMLSTv1.0) | MLST v.2.22.0 | MLST CC | ATB | Genomic resistance pattern  (STARAMR and AMRFINDER) | Circularizzed plasmid (*) |
| --- | --- | --- | --- | --- | --- | --- | --- | --- | --- | --- | --- | --- |
| 9568 | *C. coli* | faeces | 2013 | 1691434 | 215194 | 30 | cgST-13890 | 832 | ST-828 CC | CipTE | GyrA (T86I), *tet*(O)*,* 23S *(*A2075G) | - |
| 9581 | *C. coli* | faeces | 2014 | 1666249 | 136923 | 53 | cgST-20838 | 5150 | nd | CipT | GyrA (T86I), *tet*(O/32/O)*, bla*_OXA-489_ | **p9581** |
| 9567 | *C. coli* | faeces | 2014 | 1660926 | 218901 | 30 | cgST-35299 | 7159 | ST-828 CC | CipT | GyrA (T86I)*, tet*(O), *bla*_OXA-579_ | - |
| 9580 | *C. coli* | faeces | 2014 | 1690194 | 226340 | 31 | cgST-5328 | 7159 | ST-828 CC | CipTE | GyrA (T86I)*, tet*(O), *bla*_OXA-579_ | **p9580** |
| 9574 | *C. coli* | faeces | 2014 | 1743537 | 237000 | 33 | cgST-44454 | 10327 | ST-828 CC | CipTE | GyrA (T86I), *tet*(O), 23S (A2075G), 50S_L22 (A103V), *bla*_OXA-193_ | **p9574** |
| 9572 | *C. coli* | faeces | 2015 | 1735058 | 266185 | 13 | cgST-1548 | 825 | ST-828 CC | CipTE | GyrA (T86I), *tet*(O), 23S (A2075G) | **p9572** |
| 9562 | *C. coli* | faeces | 2015 | 1696281 | 384318 | 27 | cgST-18124 | 832 | ST-828 CC | CipTE | GyrA (T86I), *tet*(O), 23S (A2075G), *bla*_OXA-595_ | **p9562** |
| 13770 | *C. coli* | faeces | 2015 | 1734834 | 205267 | 34 | cgST-40159 | 1055 | ST-828 CC | Cip | GyrA (T86I), *bla*_OXA-193,_ 50S_L22 (A103V) | - |
| 13777 | *C. coli* | faeces | 2015 | 1802158 | 163084 | 62 | cgST-2593 | 1055 | ST-828 CC | CipT | GyrA (T86I), *tet*(O), *bla*_OXA-193_*,* 50S_L22 (A103V) | - |
| 13778 | *C. coli* | faeces | 2015 | 1757333 | 189755 | 99 | cgST-12640 | 1585 | ST-828 CC | CipT | GyrA (T86I), *tet*(O), *bla*_OXA-452_ | - |
| 9560 | *C. coli* | blood | 2015 | 1688909 | 226291 | 27 | cgST-35299 | 7159 | ST-828 CC | CipTE | GyrA (T86I, D90N), *tet*(O), 23S (A2075G), *bla*_OXA-193_ | **p9560** |
| 9576 | *C. coli* | faeces | 2015 | 1695231 | 227354 | 26 | cgST-35299 | 7159 | ST-828 CC | CipTE | GyrA (T86I), *tet*(O), 23S (A2075G), *bla*_OXA-193_ | **p9576** |
| 9577 | *C. coli* | faeces | 2015 | 1693421 | 162330 | 34 | cgST-35299 | 7159 | ST-828 CC | CipTE | GyrA (T86I), *tet*(O), 23S (A2075G), *bla*_OXA-193_ | **p9577** |
| 13776 | *C. coli* | faeces | 2015 | 1658748 | 218473 | 23 | cgST-4122/cgST-11833 | 8195 | ST-828 CC | CipT | GyrA (T86I), *tet*(O/32/O), *bla*_OXA-595_*, aadE* | - |
| 13786 | *C. coli* | faeces | 2016 | 1714900 | 169975 | 17 | cgST-356 | 827 | ST-828 CC | CipT | GyrA (T86I), *tet*(O), *bla*_OXA-489_*,* 50S_L22 (A103V), *aadE-Cc, aph(3')-IIIa*Δ | **p13786*** |
| 13792 | *C. coli* | faeces | 2016 | 1702156 | 166614 | 37 | cgST-13025 | 828 | ST-828 CC | CipTE | GyrA (T86I), *tet*(O), 23S (A2075G) | - |
| 13794 | *C. coli* | faeces | 2016 | 1705763 | 387524 | 30 | cgST-7797 | 832 | ST-828 CC | CipT | GyrA (T86I), *tet*(O), *bla*_OXA-193_ | - |
| 13780 | *C. coli* | faeces | 2016 | 1711931 | 216373 | 52 | cgST-69 | 9265 | ST-828 CC | CipT | GyrA (T86I), *tet*(O), *bla*_OXA-584_ | **p13780** |
| 9564 | *C. coli* | faeces | 2017 | 1726089 | 251647 | 36 | cgST-13890 | 832 | ST-828 CC | CipTE | GyrA (T86I), *tet*(O), 23S (A2075G), *bla*_OXA-584_ | - |
| 586170 | *C. coli* | faeces | 2017 | 1701676 | 170154 | 39 | cgST-2223 | 12070 | nd | Suscept. | *-* | - |
| 586173 | *C. coli* | faeces | 2017 | 1663185 | 256902 | 19 | cgST-3307 | 827 | ST-828 CC | Cip | *bla*_OXA-489,_ 50S_L22 (A103V) | - |
| 15796149 | *C. coli* | faeces | 2018 | 1655877 | 234245 | 18 | cgST-14049 | 10903 | nd | Suscept. | *bla*_OXA-452_ | - |
| 19565112 | *C. coli* | faeces | 2018 | 1742849 | 223198 | 31 | cgST-34522 | 7159 | ST-828 CC | CipTE | GyrA (T86I), *tet*(O), 23S (A2075G), *bla*_OXA-193_ | **p19565112** |
| 16979918 | *C. coli* | blood | 2019 | 1615689 | 285506 | 19 | cgST-38382 | 12068 | ST-1150 CC | CipT | GyrA (T86I), *tet*(W), *bla*_OXA-584_ | **p16979918** |
| 169799140 | *C. coli* | faeces | 2019 | 1656796 | 278789 | 21 | cgST-38232 | 3777 | ST-828 CC | T | *tet*(O), *bla*_OXA-193_ | - |
| 169799118 | *C. coli* | faeces | 2019 | 1670924 | 391726 | 7 | cgST-105 | 827 | ST-828 CC | CipT | GyrA (T86I), *tet*(O), *bla*_OXA-489,_ 50S_L22 (A103V) | - |
| 300241123 | *C. coli* | faeces | 2020 | 1795165 | 214359 | 64 | cgST-44373 | 1016 | ST-828 CC | CipTE | GyrA (T86I), *tet*(O), 23S (A2075G), *aadE-Cc* | **p300241123** |
| 3364919 | *C. coli* | faeces | 2021 | 1653822 | 144729 | 29 | cgST-13890 | 1628 | ST-828 CC | CipT | GyrA (T86I), *tet*(O)*, bla*_OXA-452,_ 50S_L22 (A103V) | - |
| 33649114 | *C. coli* | faeces | 2021 | 1643505 | 255593 | 33 | cgST-20838 | 5150 | nd | CipT | GyrA (T86I), *tet*(O/32/O)*, bla*_OXA-489_ | - |
| 33649119 | *C. coli* | faeces | 2021 | 1640487 | 213884 | 35 | cgST-20838 | 5150 | nd | CipT | GyrA (T86I), *tet*(O/32/O), *bla*_OXA-489_ | - |
| 33649110 | *C. coli* | faeces | 2021 | 1725308 | 162378 | 32 | cgST-34528/cgST-34529 | 830 | ST-828 CC | Cip | GyrA (T86I) | **p33649110** |
| 9582 | *C. jejuni* | faeces | 2013 | 1620328 | 992577 | 18 | cgST-1051 | 658 | ST-658 CC | Cip | GyrA (T86I), *bla*_OXA-193_ | - |
| 9578 | *C. jejuni* | faeces | 2013 | 1623532 | 188192 | 35 | cgST-115 | 822 | ST-21 CC | Cip | GyrA (T86I), *bla*_OXA-580_ | - |
| 9559 | *C. jejuni* | faeces | 2013 | 1755592 | 227941 | 53 | cgST-19481 | 5102 | ST-464 CC | CipT | GyrA (T86I), *tet*(O/32/O), *bla*_OXA-466,_ 50S_L22 (A103V) | - |
| 9579 | *C. jejuni* | faeces | 2014 | 1701638 | 219758 | 27 | cgST-2720 | 21 | ST-21 CC | CipT | GyrA (T86I), *tet*(O), *bla*_OXA-193_ | **p9579*** |
| 13763 | *C. jejuni* | faeces | 2014 | 1689690 | 187054 | 23 | cgST-468/cgST-474 | 4717 | nd | CipT | GyrA (T86I), *tet*(O), *bla*_OXA-193_ | - |
| 13767 | *C. jejuni* | faeces | 2015 | 1661620 | 153948 | 31 | cgST-35232 | 19 | ST-21 CC | Cip | GyrA (T86I), *bla*_OXA-193_ | - |
| 13765 | *C. jejuni* | faeces | 2015 | 1664174 | 189405 | 24 | cgST-18378 | 21 | ST-21 CC | Cip | GyrA (T86I), *bla*_OXA-193_ | - |
| 13772 | *C. jejuni* | faeces | 2015 | 1817235 | 397467 | 33 | cgST-12804 | 122 | ST-206 CC | CipT | GyrA (T86I), *tet*(O), *bla*_OXA-193_ | - |
| 13798 | *C. jejuni* | blood | 2015 | 1703099 | 162403 | 42 | cgST-11062 | 2116 | ST-353 CC | Cip | GyrA (T86I), *bla*_OXA-193_ | - |
| 13766 | *C. jejuni* | faeces | 2015 | 1788120 | 162480 | 50 | cgST-27099 | 2116 | ST-353 CC | CipTE | *GyrA* (T86I), *tet(O/32/O), 23S* (A2075G), *bla*_OXA-193_ | - |
| 13775 | *C. jejuni* | faeces | 2015 | 1746377 | 498191 | 29 | cgST-3020 | 2863 | ST-354 CC | Cip | GyrA (T86I), *tet*(O), *bla*_OXA-466_*,* 50S_L22 (A103V) | - |
| 13768 | *C. jejuni* | faeces | 2015 | 1696035 | 274116 | 36 | cgST-10191 | 7991 | nd | CipT | GyrA (T86I), *tet*(O), *bla*_OXA-630_ | - |
| 13764 | *C. jejuni* | faeces | 2015 | 1658638 | 208343 | 15 | cgST-39618/cgST-434827 | 9354 | nd | Cip | GyrA (T86I), *bla*_OXA-449_ | - |
| 13762 | *C. jejuni* | faeces | 2015 | 1730716 | 176752 | 45 | cgST-39287 | 10039 | ST-403 CC | Suscept. | *-* | - |
| 13785 | *C. jejuni* | faeces | 2016 | 1721602 | 242480 | 36 | cgST-14318 | 19 | ST-21 CC | CipT | GyrA (T86I), *tet*(O), *bla*_OXA-193_ | - |
| 13788 | *C. jejuni* | faeces | 2016 | 1660033 | 167344 | 23 | cgST-18378 | 21 | ST-21 CC | Cip | GyrA (T86I), *bla*_OXA-193_ | - |
| 13787 | *C. jejuni* | faeces | 2016 | 1627649 | 1002525 | 17 | cgST-9169 | 49 | ST-49 CC | T | *tet*(O/32/O), *bla*_OXA-461_ | - |
| 13790 | *C. jejuni* | faeces | 2016 | 1740024 | 189454 | 95 | cgST-10789 | 50 | ST-21 CC | CipTGm | GyrA (T86I), *bla*_OXA-193_*, aadE, aph(3')-IIIa* | - |
| 13784 | *C. jejuni* | faeces | 2016 | 1681236 | 155823 | 34 | cgST-5849 | 50 | ST-21 CC | Suscept. | *bla*_OXA-451_ | **p13784** |
| 13793 | *C. jejuni* | faeces | 2016 | 1722064 | 257370 | 39 | cgST-3893 | 161 | ST-52 CC | CipT | GyrA (T86I), *tet*(O), *bla*_OXA-193_ | - |
| 13789 | *C. jejuni* | faeces | 2016 | 1684969 | 214171 | 35 | cgST-3106 | 257 | ST-257 CC | CipT | GyrA (T86I), *tet*(O), *bla*_OXA-193_ | - |
| 13781 | *C. jejuni* | faeces | 2016 | 1746387 | 292545 | 32 | cgST-24679 | 1039 | nd | Suscept. | *bla*_OXA-193_ | - |
| 13797 | *C. jejuni* | faeces | 2016 | 1777624 | 162252 | 38 | cgST-27099 | 2116 | ST-353 CC | CipTE | GyrA (T86I), *tet*(O/32/O), 23S (A2075G), *bla*_OXA-193_ | - |
| 13783 | *C. jejuni* | faeces | 2016 | 1678928 | 389566 | 18 | cgST-37298 | 3335 | ST-206 CC | CipT | GyrA (T86I), *tet*(O/32/O), *bla*_OXA-193_ | - |
| 13791 | *C. jejuni* | faeces | 2016 | 1645911 | 357098 | 17 | cgST-37931 | 3335 | ST-206 CC | CipT | GyrA (T86I), *tet*(O/32/O), *bla*_OXA-193_ | - |
| 13796 | *C. jejuni* | faeces | 2016 | 1668892 | 159975 | 23 | cgST-37931 | 3335 | ST-206 CC | CipT | GyrA (T86I), *tet*(O/32/O), *bla*_OXA-193_ | - |
| 13782 | *C. jejuni* | faeces | 2016 | 1666024 | 189410 | 34 | cgST-6525 | 5018 | ST-21 CC | Suscept. | *bla*_OXA-581_ | - |
| 833116 | *C. jejuni* | faeces | 2016 | 1687428 | 161747 | 29 | cgST-35217/cgST-35432/cgST-35608 | 122 | ST-206 CC | CipT | GyrA (T86I), *tet*(O/32/O), *bla*_OXA-193_ | - |
| 833112 | *C. jejuni* | faeces | 2016 | 1781064 | 152251 | 37 | cgST-10209 | 22 | ST-22 CC | CipT | GyrA (T86I), *tet*(O), *bla*_OXA-193_ | p833112_4kb, p833122_26kb, p833112_43kb* |
| 9565 | *C. jejuni* | faeces | 2017 | 1635426 | 157420 | 38 | cgST-20262/37291 | 227 | ST-206 CC | Suscept. | *bla*_OXA-193_ | - |
| 9573 | *C. jejuni* | faeces | 2017 | 1620029 | 449909 | 16 | cgST-16869 | 658 | ST-658 CC | Cip | GyrA (T86I), *bla*_OXA-193_ | - |
| 9571 | *C. jejuni* | faeces | 2017 | 1742279 | 154555 | 35 | cgST-14056 | 1039 | nd | Cip | GyrA (T86I), *bla*_OXA-193_ | - |
| 9563 | *C. jejuni* | faeces | 2017 | 1611835 | 285489 | 17 | cgST-2138 | 1044 | ST-658 CC | Suscept. | *bla*_OXA-630_ | - |
| 9569 | *C. jejuni* | faeces | 2017 | 1622568 | 465822 | 13 | cgST-14496 | 1721 | nd | Suscept. | *bla*_OXA-193_ | - |
| 9566 | *C. jejuni* | faeces | 2017 | 1672421 | 218206 | 26 | cgST-20652 | 1943 | ST-21 CC | Suscept. | *bla*_OXA-193_ | - |
| 7587111 | *C. jejuni* | faeces | 2017 | 1622136 | 992015 | 20 | cgST-2138 | 1044 | ST-658 CC | Suscept. | *bla*_OXA-193_ | - |
| 586168 | *C. jejuni* | faeces | 2017 | 1721925 | 160983 | 31 | cgST-35167 | 122 | ST-206 CC | Suscept. | *bla*_OXA-193_ | - |
| 586174 | *C. jejuni* | faeces | 2017 | 1676262 | 153955 | 45 | cgST-19995 | 19 | ST-21 CC | Cip | GyrA (T86I), *bla*_OXA-193_*,* 50S_L22 (A103V) | - |
| 586167 | *C. jejuni* | faeces | 2017 | 1665935 | 154480 | 27 | cgST-18182 | 21 | ST-21 CC | Suscept. | *bla*_OXA-193_ | - |
| 586177 | *C. jejuni* | faeces | 2017 | 1654084 | 185336 | 39 | cgST-14496 | 2861 | nd | Cip | GyrA (T86I), *bla*_OXA-193_ | - |
| 1956516 | *C. jejuni* | faeces | 2017 | 1744359 | 334769 | 40 | cgST-3020 | 2863 | ST-354 CC | CipT | GyrA (T86I), *tet*(O), *bla*_OXA-466_*,* 50S_L22 (A103V) | - |
| 586165 | *C. jejuni* | faeces | 2017 | 1743276 | 277311 | 19 | cgST-401 | 3076 | ST-658 CC | T | GyrA (T86A), *tet*(O), *bla*_OXA-193_ | - |
| 15796145 | *C. jejuni* | faeces | 2018 | 1599139 | 84463 | 33 | cgST-35030 | 12069 | nd | Suscept. | *-* | - |
| 1956515 | *C. jejuni* | faeces | 2018 | 1686364 | 90318 | 52 | cgST-21697 | 122 | ST-206 CC | CipT | GyrA (T86I), *tet*(O/32/O), *bla*_OXA-193_ | - |
| 15796144 | *C. jejuni* | faeces | 2018 | 1647232 | 114636 | 32 | cgST-19995 | 19 | ST-21 CC | Cip | GyrA (T86I), *bla*_OXA-193_ | - |
| 19565110 | *C. jejuni* | faeces | 2018 | 1647728 | 153955 | 37 | cgST-19995 | 19 | ST-21 CC | Cip | GyrA (T86I), *bla*_OXA-193_*,* 50S_L22 (A103V) | - |
| 1956512 | *C. jejuni* | blood | 2018 | 1737652 | 202144 | 31 | cgST-5040 | 1962 | nd | CipT | GyrA (T86I), *tet*(O/32/O), *bla*_OXA-499_ | - |
| 1956518 | *C. jejuni* | faeces | 2018 | 1655180 | 325140 | 16 | cgST-10204/cgST-23931 | 2254 | ST-257 CC | CipT | GyrA (T86I)*, tet*(O), *bla*_OXA-461_ | - |
| 1956517 | *C. jejuni* | faeces | 2018 | 1759630 | 335225 | 33 | cgST-20941/cgST-36126/cgST-36692 | 2274 | ST-257 CC | CipT | GyrA (T86V)*, tet*(O), *bla*_OXA-193_ | - |
| 15796147 | *C. jejuni* | faeces | 2018 | 1962823 | 211545 | 76 | cgST-1257 | 2850 | ST-446 CC | CipT | GyrA (T86I)*, tet*(O), *bla*_OXA-193_ | - |
| 15796146 | *C. jejuni* | faeces | 2018 | 1644020 | 58145 | 68 | cgST-37931 | 3335 | ST-206 CC | CipT | GyrA (T86I)*, tet*(O/32/O), *bla*_OXA-193_ | - |
| 15796152 | *C. jejuni* | blood | 2018 | 1612279 | 175951 | 36 | cgST-19109 | 49 | ST-49 CC | Cip | GyrA (T86I)*, bla*_OXA-461_ | - |
| 15796151 | *C. jejuni* | faeces | 2018 | 1700079 | 104456 | 46 | cgST-7704/cgST-7710 | 50 | ST-21 CC | Cip | GyrA (T86I)*, bla*_OXA-193_ | - |
| 15796153 | *C. jejuni* | faeces | 2018 | 1636102 | 94583 | 35 | cgST-2703 /cgST-4169/cgST-4202 /cgST-35247 | 50 | ST-21 CC | Cip | GyrA (T86I)*, bla*_OXA-193_ | - |
| 15796150 | *C. jejuni* | faeces | 2018 | 1726877 | 156980 | 42 | cgST-13166 | 50 | ST-21 CC | CipT | GyrA (T86I)*, tet*(O), *bla*_OXA-193_ | **p15796150*** |
| 19565113 | *C. jejuni* | faeces | 2018 | 1719055 | 161058 | 29 | cgST-1997/cgST-24929 | 50 | ST-21 CC | CipT | GyrA (T86I)*, tet*(O/32/O), *bla*_OXA-193_ | - |
| 15796148 | *C. jejuni* | faeces | 2018 | 1675252 | 133804 | 26 | cgST-16099 | 572 | ST-206 CC | Cip | GyrA (T86I)*, bla*_OXA-193_ | - |
| 169799143 | *C. jejuni* | faeces | 2019 | 1653357 | 294639 | 17 | cgST-6159 | 3720 | ST-49 CC | Cip | GyrA (T86I)*, tet*(O), *bla*_OXA-193_ | - |
| 16979914 | *C. jejuni* | faeces | 2019 | 1713132 | 240613 | 30 | cgST-21422 | 2364 | ST-353 CC | Cip | GyrA (T86I) | - |
| 169799144 | *C. jejuni* | faeces | 2019 | 1647232 | 114636 | 32 | cgST-14496 | 2861 | nd | CipTE | GyrA (T86I)*, bla*_OXA-193_ | - |
| 169799131 | *C. jejuni* | faeces | 2019 | 1677539 | 153924 | 19 | cgST-15013 | 3335 | ST-206 CC | CipT | GyrA (T86I)*, tet*(O/32/O), *bla*_OXA-193_ | - |
| 169799116 | *C. jejuni* | faeces | 2019 | 1676151 | 153924 | 19 | cgST-37298 | 3335 | ST-206 CC | T | *tet*(O/32/O), *bla*_OXA-193_ | - |
| 169799112 | *C. jejuni* | faeces | 2019 | 1722236 | 248212 | 39 | cgST-3662 | 354 | ST-354 CC | CipT | GyrA (T86I)*, tet*(O), *bla*_OXA-61_ | - |
| 169799119 | *C. jejuni* | faeces | 2019 | 1736792 | 286969 | 34 | cgST-18363 | 400 | ST-353 CC | E | GyrA (T86I)*, tet*(O/32/O), *bla*_OXA-452_*,* 50S_L22 (A103V) | - |
| 169799139 | *C. jejuni* | faeces | 2019 | 1639314 | 138666 | 26 | cgST-18147 | 50 | ST-21 CC | CipTEGm | GyrA (T86I)*, bla*_OXA-193_ | - |
| 169799111 | *C. jejuni* | faeces | 2019 | 1674476 | 153917 | 19 | cgST-16099 | 572 | ST-206 CC | CipT | GyrA (T86I)*, bla*_OXA-193_ | - |
| 169799129 | *C. jejuni* | faeces | 2019 | 1742831 | 181373 | 32 | cgST-2440 | 824 | ST-257 CC | CipT | GyrA (T86I)*, tet*(O), 50S_L22 (A103V) | - |
| 54_9_20 | *C. jejuni* | faeces | 2020 | 1675325 | 190088 | 24 | cgST-36045 | 21 | ST-21 CC | CipT | GyrA (T86I)*, tet*(O), *bla*_OXA-193_ | - |
| 43_1_20 | *C. jejuni* | faeces | 2020 | 1602635 | 154126 | 18 | cgST-35030 | 45 | ST-45 CC | Suscept. | *bla*_OXA-193_ | - |
| 53_1_20 | *C. jejuni* | faeces | 2020 | 1647572 | 153880 | 18 | cgST-37180/cgST-37298 | 335 | ST-607 CC | CipT | GyrA (T86I)*, tet*(O/32/O), *bla*_OXA-193_ | - |
| 54_3_20 | *C. jejuni* | faeces | 2020 | 1645687 | 114766 | 47 | cgST-37180/cgST-37298 | 335 | ST-607 CC | T | *tet*(O/32/O), *bla*_OXA-193_ | - |
| 36_1_20 | *C. jejuni* | faeces | 2020 | 1705885 | 254245 | 15 | cgST-3662 | 354 | ST-354 CC | CipT | GyrA (T86I)*, tet*(O), *bla*_OXA-61_ | - |
| 48_2_20 | *C. jejuni* | faeces | 2020 | 1674198 | 153873 | 17 | cgST-16099 | 572 | ST-206 CC | Cip | GyrA (T86I)*, bla*_OXA-193_ | - |
| 55_38_20 | *C. jejuni* | faeces | 2020 | 1699085 | 159692 | 40 | cgST-24430 | 822 | ST-21 CC | Cip | GyrA (T86I)*, bla*_OXA-193_ | - |
| 36_3_20 | *C. jejuni* | faeces | 2020 | 1747964 | 154511 | 29 | cgST-14056 | 1039 | nd | Cip | GyrA (T86I)*, bla*_OXA-193_ | - |
| 36_2_20 | *C. jejuni* | faeces | 2020 | 1721582 | 110513 | 38 | cgST-14999 | 2066 | ST-52 CC | CipT | GyrA (T86I)*, tet*(O) | - |
| 38_1_20 | *C. jejuni* | faeces | 2020 | 1722786 | 110512 | 37 | cgST-14999 | 2066 | ST-52 CC | CipT | GyrA (T86I)*, tet*(O) | - |
| 31_3_20 | *C. jejuni* | faeces | 2020 | 1765844 | 156245 | 37 | cgST-11062 | 2116 | ST-353 CC | Cip | GyrA (T86I)*, bla*_OXA-193_ | - |
| 50_1_20 | *C. jejuni* | faeces | 2020 | 1771330 | 156556 | 36 | cgST-11062 | 2116 | ST-353 CC | Cip | GyrA (T86I)*, bla*_OXA-193_ | **p50_1_20** |
| 55_36_20 | *C. jejuni* | faeces | 2020 | 1811001 | 156245 | 33 | cgST-11062 | 2116 | ST-353 CC | Cip | GyrA (T86I)*, bla*_OXA-193_ | - |
| 55_35_20 | *C. jejuni* | faeces | 2020 | 1654609 | 328588 | 12 | cgST-38159 | 2133 | nd | Suscept. | *bla*_OXA-61_ | - |
| 54_8_20 | *C. jejuni* | faeces | 2020 | 1866163 | 148705 | 47 | cgST-19570 | 2844 | ST-460 CC | Cip | GyrA (T86I)*,* 50S_L22 (A103V) | - |
| 38_2_20 | *C. jejuni* | faeces | 2020 | 1747920 | 402644 | 28 | cgST-3020 | 2863 | ST-354 CC | CipT | GyrA (T86I)*, tet*(O), *bla*_OXA-466_*,* 50S_L22 (A103V) | - |
| 55_32_20 | *C. jejuni* | faeces | 2020 | 1756031 | 211058 | 61 | cgST-3020 | 2863 | ST-354 CC | CipT | GyrA (T86I)*, tet*(O), *bla*_OXA-466_*,* 50S_L22 (A103V) | - |
| 36_4_20 | *C. jejuni* | faeces | 2020 | 1737557 | 153880 | 29 | cgST-37298 | 3335 | ST-206 CC | CipT | GyrA (T86I)*, tet*(O/32/O), *bla*_OXA-193_ | - |
| 55_39_20 | *C. jejuni* | faeces | 2020 | 1616393 | 189253 | 23 | cgST-39618 | 9354 | nd | Cip | GyrA (T86I)*, bla*_OXA-449_ | - |
| 35_2_20 | *C. jejuni* | faeces | 2020 | 1750752 | 181929 | 33 | cgST-3020 | 11200 | nd | CipT | GyrA (T86I)*, tet*(O), *bla*_OXA-466_*,* 50S_L22 (A103V) | - |
| 3364918 | *C. jejuni* | faeces | 2021 | 1615689 | 285506 | 19 | cgST-2138/cgST-8697 | 1044 | ST-658 CC | Suscept. | *bla*_OXA-61_ | - |
| 33649133 | *C. jejuni* | faeces | 2021 | 1727333 | 161747 | 24 | cgST-1644 | 122 | ST-206 CC | CipT | GyrA (T86I)*, tet*(O/32/O), *bla*_OXA-193_ | - |
| 3364914 | *C. jejuni* | faeces | 2021 | 1649967 | 153955 | 26 | cgST-19995 | 19 | ST-21 CC | Cip | GyrA (T86I)*, bla*_OXA-193_, 50S_L22 (A103V) | - |
| 33649138 | *C. jejuni* | faeces | 2021 | 1768774 | 156290 | 39 | cgST-11062 | 2116 | ST-353 CC | Cip | GyrA (T86I)*, bla*_OXA-193_ | **p33649138** |
| 33649122 | *C. jejuni* | faeces | 2021 | 1745037 | 334771 | 31 | cgST-3020 | 2863 | ST-354 CC | CipT | GyrA (T86I)*, tet(O), bla*_OXA-466_*,* 50S_L22 (A103V) | - |
| 33649123 | *C. jejuni* | faeces | 2021 | 1744461 | 334746 | 28 | cgST-19263 | 2863 | ST-354 CC | CipT | GyrA (T86I)*, tet*(O), *bla*_OXA-466_*,* 50S_L22 (A103V) | - |
| 33649121 | *C. jejuni* | faeces | 2021 | 1708097 | 254270 | 21 | cgST-3662 | 354 | ST-354 CC | CipT | GyrA (T86I)*,* tet(O), *bla*_OXA-460_ | - |
| 33649141 | *C. jejuni* | faeces | 2021 | 1625365 | 288923 | 19 | cgST-10201 | 3720 | ST-49 CC | Suscept. | *bla*_OXA-193_ | - |
| 33649143 | *C. jejuni* | faeces | 2021 | 1665508 | 337954 | 23 | cgST-15693 | 38 | ST-48 CC | Cip | GyrA (T86I)*, bla*_OXA-193_ | - |
| 3364915 | *C. jejuni* | faeces | 2021 | 1657378 | 188196 | 14 | cgST-13875/cgST-15969/cgST-15972/cgST-16981 | 50 | ST-21 CC | CipT | GyrA (T86I)*, tet*(O), *bla*_OXA-193_ | - |
| 33649117 | *C. jejuni* | faeces | 2021 | 1623394 | 188197 | 14 | cgST-13875/cgST-15969/cgST-16981 | 50 | ST-21 CC | CipT | GyrA (T86I)*, tet*(O), *bla*_OXA-193_ | - |
| 33649116 | *C. jejuni* | faeces | 2021 | 1780023 | 112018 | 50 | cgST-15988/cgST-44539/cgST-44555/cgST-44558 | 6175 | ST-21 CC | CipT | GyrA (T86I), *tet*(O), *bla*_OXA-193_ | - |
| 33649125 | *C. jejuni* | faeces | 2021 | 1714744 | 194126 | 36 | cgST-4628/cgST-15226 | 6461 | ST-353 CC | CipT | GyrA (T86I), *tet*(O/32/O), *bla*_OXA-193_*,* 50S_L22 (A103V), *aadE* | - |
| 33649128 | *C. jejuni* | faeces | 2021 | 1646206 | 144327 | 31 | cgST-1926/cgST-5097/cgST-5155 | 677 | ST-677 CC | Suscept. | *bla*_OXA-447_ | - |
| 33649118 | *C. jejuni* | faeces | 2021 | 1664093 | 159438 | 24 | cgST-24430 | 822 | ST-21 CC | Suscept. | *bla*_OXA-193_ | - |

*: Plasmids coding for Tet(O); Suscept.: Susceptible to Cip, T, E and Gm; Δ: partial gene.
