# Supplementary material for: Antibiotic resistance, plasmids, and virulence-associated markers in human strains of Campylobacter jejuni and Campylobacter coli isolated in Italy
Source: Front Microbiol. 2024 Jan 8;14:1293666. doi: 10.3389/fmicb.2023.1293666 (PMC10800408; doi:10.3389/fmicb.2023.1293666)
Supplement: Supplementary file 3 [file Table_3.DOCX]

**Table S3**. Virulence Factors associated with bacterial species different from *Campylobacter* spp.

| **Strain** | ***Campylobacter* specie** | **MLST (CC)** | **MLST (ST)** | **Antiphagocytosis^a^** | **Serum resistance and immune evastion^a^** | **Others^a^** | **Secretion system^a^** | | | | **Toxin^a^** |
| --- | --- | --- | --- | --- | --- | --- | --- | --- | --- | --- | --- |
|  |  |  |  | **Capsule**  **(*Klebsiella* spp.)^b^** | **LPS**  **(*Francisella* spp.) ^b^** | **O-antigen (*Yersinia* spp.) ^b^** | **ACE T6SS (*Escherichia coli*)**  *aec17* **^b^** | **T6SS (*Aeromonas* spp.) ^b^** | **VAS effector proteins (*Vibrio* spp.)**  *hcp-2* **^b^** | **Lvh (*Legionella* spp. vir homologs) type IVA secretion system**  *virB* **^b^** | **Phytotoxin phaseolotoxin (*Pseudomonas* spp.)**  *cysC1* **^b^** |
| 55_32_20 | *C. jejuni* | 354 | 2863 | - | - | - | + | + | + | - | - |
| 1956516 | *C. jejuni* | 354 | 2863 | - | - | - | + | + | + | - | - |
| 38_2_20 | *C. jejuni* | 354 | 2863 | - | - | - | + | + | + | - | - |
| 33649122 | *C. jejuni* | 354 | 2863 | - | - | - | + | + | + | - | - |
| 13775 | *C. jejuni* | 354 | 2863 | - | - | - | + | + | + | - | - |
| 33649123 | *C. jejuni* | 354 | 2863 | - | - | - | + | + | + | - | - |
| 38_1_20 | *C. jejuni* | 52 | 2066 | - | - | - | + | + | + | - | + |
| 36_2_20 | *C. jejuni* | 52 | 2066 | - | - | - | + | + | + | - | - |
| 13793 | *C. jejuni* | 52 | 161 | - | - | - | + | + | + | - | - |
| 1956517 | *C. jejuni* | 257 | 2274 | - | - | - | + | + | + | - | - |
| 169799129 | *C. jejuni* | 257 | 824 | - | - | - | + | + | + | - | - |
| 300241123 | *C. coli* | 828 | 1016 | - | - | - | + | + | + | - | - |
| 33649116 | *C. jejuni* | 21 | 6175 | - | - | - | + | + | + | - | - |
| 33649119 | *C. coli* | ND | 5150 | - | - | - | + | + | + | - | - |
| 13781 | *C. jejuni* | ND | 1039 | - | - | + | + | + | + | - | - |
| 9571 | *C. jejuni* | ND | 1039 | - | - | - | + | + | + | - | + |
| 36_3_20 | *C. jejuni* | ND | 1039 | - | - | + | + | + | + | - | - |
| 13762 | *C. jejuni* | 403 | 10039 | - | - | - | + | + | + | - | - |
| 13768 | *C. jejuni* | ND | 7991 | - | - | - | + | + | + | - | + |
| 9559 | *C. jejuni* | 464 | 5102 | - | - | - | + | + | + | - | - |
| 35_2_20 | *C. jejuni* | ND | 11200 | - | - | - | + | + | + | - | - |
| 169799119 | *C. jejuni* | 353 | 400 | - | - | - | + | + | + | - | - |
| 33649125 | *C. jejuni* | 353 | 6461 | - | - | - | + | + | + | - | - |
| 13798 | *C. jejuni* | 353 | 2116 | - | - | - | + | + | + | - | - |
| 31_3_20 | *C. jejuni* | 353 | 2116 | - | - | - | + | + | + | + | - |
| 13797 | *C. jejuni* | 353 | 2116 | - | - | - | + | + | + | + | - |
| 13766 | *C. jejuni* | 353 | 2116 | - | - | - | + | + | + | + | - |
| 50_1_20 | *C. jejuni* | 353 | 2116 | - | - | - | + | + | + | + | - |
| 33649138 | *C. jejuni* | 353 | 2116 | - | - | - | + | + | + | + | - |
| 55_36_20 | *C. jejuni* | 353 | 2116 | - | - | - | + | + | + | + | - |
| 15796147 | *C. jejuni* | 446 | 2850 | - | - | - | + | + | + | + | - |
| 54_8_20 | *C. jejuni* | 460 | 2844 | - | - | - | + | + | + | + | + |
| 33649110 | *C. coli* | 828 | 830 | - | - | - | - | - | - | + | + |
| 9564 | *C. coli* | 828 | 832 | - | - | - | - | - | - | + | + |
| 13780 | *C. coli* | 828 | 9265 | - | - | - | - | - | - | + | - |
| 9580 | *C. coli* | 828 | 7159 | + | + | + | - | - | - | + | - |
| 9577 | *C. coli* | 828 | 7159 | + | + | + | - | - | - | + | - |
| 9576 | *C. coli* | 828 | 7159 | + | + | + | - | - | - | + | - |
| 9560 | *C. coli* | 828 | 7159 | + | + | + | - | - | - | + | - |
| 13777 | *C. coli* | 828 | 1055 | + | + | + | - | - | - | + | - |
| 19565112 | *C. coli* | 828 | 7159 | + | + | + | - | - | - | + | - |
| 9572 | *C. coli* | 828 | 825 | + | + | + | - | - | - | - | - |
| 9567 | *C. coli* | 828 | 7159 | + | + | + | - | - | - | - | - |
| 13776 | *C. coli* | 828 | 8195 | + | + | - | - | - | - | - | - |
| 169799140 | *C. coli* | 828 | 377 | + | + | - | - | - | - | - | - |
| 9581 | *C. coli* | ND | 5150 | - | - | + | - | - | - | + | - |
| 586170 | *C. coli* | ND | 12070 | - | - | - | - | - | - | + | + |
| 833112 | *C. jejuni* | 22 | 22 | - | - | - | - | - | - | + | + |
| 15796145 | *C. jejuni* | ND | 12069 | - | - | - | - | - | - | - | - |
| 43_1_20 | *C. jejuni* | 45 | 45 | - | - | - | - | - | - | - | - |
| 55_39_20 | *C. jejuni* | ND | 9354 | - | - | - | - | - | - | - | + |
| 13764 | *C. jejuni* | ND | 9354 | - | - | - | - | - | - | - | + |
| 586177 | *C. jejuni* | ND | 2861 | - | - | + | - | - | - | - | - |
| 169799144 | *C. jejuni* | ND | 2861 | - | - | + | - | - | - | - | - |
| 9569 | *C. jejuni* | ND | 1721 | - | - | + | - | - | - | - | - |
| 586173 | *C. coli* | 828 | 827 | - | - | + | - | - | - | - | + |
| 3364919 | *C. coli* | 828 | 1628 | - | - | + | - | - | - | - | - |
| 13786 | *C. coli* | 828 | 827 | - | - | + | - | - | - | - | + |
| 13778 | *C. coli* | 828 | 1585 | - | - | - | - | - | - | - | + |
| 169799118 | *C. coli* | 828 | 827 | - | - | - | - | - | - | - | + |
| 9568 | *C. coli* | 828 | 832 | - | - | - | - | - | - | - | + |
| 13794 | *C. coli* | 828 | 832 | - | - | - | - | - | - | - | + |
| 13770 | *C. coli* | 828 | 1055 | - | - | - | - | - | - | - | + |
| 586168 | *C. jejuni* | 206 | 122 | - | - | - | - | - | - | - | + |
| 9565 | *C. jejuni* | 206 | 227 | - | - | - | - | - | - | - | + |
| 833116 | *C. jejuni* | 206 | 122 | - | - | - | - | - | - | - | + |
| 33649133 | *C. jejuni* | 206 | 122 | - | - | - | - | - | - | - | + |
| 1956515 | *C. jejuni* | 206 | 122 | - | - | - | - | - | - | - | + |
| 15796149 | *C. coli* | ND | 10903 | - | - | - | - | - | - | - | + |
| 15796150 | *C. jejuni* | 21 | 50 | - | - | - | - | - | - | - | - |
| 9579 | *C. jejuni* | 21 | 21 | - | - | - | - | - | - | - | - |
| 13763 | *C. jejuni* | ND | 4717 | - | - | - | - | - | - | - | - |
| 9574 | *C. coli* | 828 | 10327 | - | - | - | - | - | - | - | - |
| 13772 | *C. jejuni* | 206 | 122 | - | - | - | - | - | - | - | - |
| 13789 | *C. jejuni* | 257 | 257 | - | - | - | - | - | - | - | - |
| 1956518 | *C. jejuni* | 257 | 2254 | - | - | - | - | - | - | - | - |
| 33649114 | *C. coli* | ND | 5150 | - | - | - | - | - | - | - | - |
| 54_9_20 | *C. jejuni* | 21 | 21 | - | - | - | - | - | - | - | - |
| 55_38_20 | *C. jejuni* | 21 | 822 | - | - | - | - | - | - | - | - |
| 9566 | *C. jejuni* | 21 | 1923 | - | - | - | - | - | - | - | - |
| 586167 | *C. jejuni* | 21 | 21 | - | - | - | - | - | - | - | - |
| 3364915 | *C. jejuni* | 21 | 50 | - | - | - | - | - | - | - | - |
| 13782 | *C. jejuni* | 21 | 5018 | - | - | - | - | - | - | - | - |
| 33649118 | *C. jejuni* | 21 | 822 | - | - | - | - | - | - | - | - |
| 13765 | *C. jejuni* | 21 | 21 | - | - | - | - | - | - | - | - |
| 19565113 | *C. jejuni* | 21 | 50 | - | - | - | - | - | - | - | - |
| 3364914 | *C. jejuni* | 21 | 19 | - | - | - | - | - | - | - | - |
| 9578 | *C. jejuni* | 21 | 822 | - | - | - | - | - | - | - | - |
| 15796151 | *C. jejuni* | 21 | 50 | - | - | - | - | - | - | - | - |
| 19565110 | *C. jejuni* | 21 | 19 | - | - | - | - | - | - | - | - |
| 13784 | *C. jejuni* | 21 | 50 | - | - | - | - | - | - | - | - |
| 15796153 | *C. jejuni* | 21 | 50 | - | - | - | - | - | - | - | - |
| 13790 | *C. jejuni* | 21 | 50 | - | - | - | - | - | - | - | - |
| 13767 | *C. jejuni* | 21 | 19 | - | - | - | - | - | - | - | - |
| 586174 | *C. jejuni* | 21 | 19 | - | - | - | - | - | - | - | - |
| 33649117 | *C. jejuni* | 21 | 50 | - | - | - | - | - | - | - | - |
| 15796144 | *C. jejuni* | 21 | 19 | - | - | - | - | - | - | - | - |
| 169799139 | *C. jejuni* | 21 | 50 | - | - | - | - | - | - | - | - |
| 13785 | *C. jejuni* | 21 | 19 | - | - | - | - | - | - | - | - |
| 13788 | *C. jejuni* | 21 | 21 | - | - | - | - | - | - | - | - |
| 16979914 | *C. jejuni* | 353 | 2364 | - | - | - | - | - | - | - | - |
| 36_4_20 | *C. jejuni* | 206 | 3335 | - | - | - | - | - | - | - | - |
| 54_3_20 | *C. jejuni* | 206 | 3335 | - | - | - | - | - | - | - | - |
| 53_1_20 | *C. jejuni* | 206 | 3335 | - | - | - | - | - | - | - | - |
| 48_2_20 | *C. jejuni* | 206 | 572 | - | - | - | - | - | - | - | - |
| 169799116 | *C. jejuni* | 206 | 3335 | - | - | - | - | - | - | - | - |
| 169799111 | *C. jejuni* | 206 | 572 | - | - | - | - | - | - | - | - |
| 15796146 | *C. jejuni* | 206 | 3335 | - | - | - | - | - | - | - | - |
| 13791 | *C. jejuni* | 206 | 3335 | - | - | - | - | - | - | - | - |
| 13783 | *C. jejuni* | 206 | 3335 | - | - | - | - | - | - | - | - |
| 169799131 | *C. jejuni* | 206 | 3335 | - | - | - | - | - | - | - | - |
| 13796 | *C. jejuni* | 206 | 3335 | - | - | - | - | - | - | - | - |
| 15796148 | *C. jejuni* | 206 | 572 | - | - | - | - | - | - | - | - |
| 9573 | *C. jejuni* | 658 | 658 | - | - | - | - | - | - | - | - |
| 9563 | *C. jejuni* | 658 | 1044 | - | - | - | - | - | - | - | - |
| 9582 | *C. jejuni* | 658 | 658 | - | - | - | - | - | - | - | - |
| 586165 | *C. jejuni* | 658 | 3076 | - | - | - | - | - | - | - | - |
| 7587111 | *C. jejuni* | 658 | 1044 | - | - | - | - | - | - | - | - |
| 3364918 | *C. jejuni* | 658 | 1044 | - | - | - | - | - | - | - | - |
| 33649141 | *C. jejuni* | 49 | 3720 | - | - | - | - | - | - | - | - |
| 169799143 | *C. jejuni* | 49 | 3720 | - | - | - | - | - | - | - | - |
| 13787 | *C. jejuni* | 49 | 49 | - | - | - | - | - | - | - | - |
| 15796152 | *C. jejuni* | 49 | 49 | - | - | - | - | - | - | - | - |
| 13792 | *C. coli* | 828 | 828 | - | - | - | - | - | - | - | - |
| 36_1_20 | *C. jejuni* | 354 | 354 | - | - | - | - | - | - | - | - |
| 169799112 | *C. jejuni* | 354 | 354 | - | - | - | - | - | - | - | - |
| 33649121 | *C. jejuni* | 354 | 354 | - | - | - | - | - | - | - | - |
| 55_35_20 | *C. jejuni* | ND | 2133 | - | - | - | - | - | - | - | - |
| 9562 | *C. coli* | 827 | 832 | - | - | - | - | - | - | - | - |
| 33649143 | *C. jejuni* | 48 | 38 | - | - | - | - | - | - | - | - |
| 33649128 | *C. jejuni* | 677 | 677 | - | - | - | - | - | - | - | - |
| 1956512 | *C. jejuni* | ND | 1962 | - | - | - | - | - | - | - | - |
| 16979918 | *C. coli* | 1150 | 12068 | - | - | - | - | - | - | - | - |

a: Virulence Factor class; b : Virulence Factor
